# scASprofiler: profiling single-cell RNA splicing with a deep convolutional generative network

**DOI:** 10.1093/bib/bbag497

**Published:** 2026-09-11

**Authors:** Pengwei Hu, Pengcheng Song, Bingjie Dai, Chunshen Long, Hanshuang Li, Yongchun Zuo, Yongqiang Xing

**Affiliations:** State Key Laboratory of Reproductive Regulation and Breeding of Grassland Livestock, College of Life Sciences, Inner Mongolia University, No. 49, Xilin Gol South Road, Yuquan District, Hohhot 010020, China; State Key Laboratory of Reproductive Regulation and Breeding of Grassland Livestock, College of Life Sciences, Inner Mongolia University, No. 49, Xilin Gol South Road, Yuquan District, Hohhot 010020, China; State Key Laboratory of Reproductive Regulation and Breeding of Grassland Livestock, College of Life Sciences, Inner Mongolia University, No. 49, Xilin Gol South Road, Yuquan District, Hohhot 010020, China; State Key Laboratory of Reproductive Regulation and Breeding of Grassland Livestock, College of Life Sciences, Inner Mongolia University, No. 49, Xilin Gol South Road, Yuquan District, Hohhot 010020, China; State Key Laboratory of Reproductive Regulation and Breeding of Grassland Livestock, College of Life Sciences, Inner Mongolia University, No. 49, Xilin Gol South Road, Yuquan District, Hohhot 010020, China; State Key Laboratory of Reproductive Regulation and Breeding of Grassland Livestock, College of Life Sciences, Inner Mongolia University, No. 49, Xilin Gol South Road, Yuquan District, Hohhot 010020, China; Inner Mongolia Key Laboratory of Life Health and Bioinformatics, School of Life Science and Technology, Inner Mongolia University of Science and Technology, No. 7, Aerding Street, Kundulun District, Baotou 014010, China

**Keywords:** alternative splicing, scRNA-seq, VAE-GAN, imputation, cell heterogeneity

## Abstract

Single-cell RNA sequencing (scRNA-seq) enables the investigation of alternative splicing (AS) at cellular resolution. However, the analysis of AS in scRNA-seq data is constrained by sparse splice-junction coverage, a consequence of low sequencing depth per cell. This limitation is particularly pronounced in 3′-biased, droplet-based protocols. To overcome this, we developed scASprofiler, a tailored deep convolutional generative network designed to decipher AS with single-cell resolution. scASprofiler performs missing-value imputation of junction read counts by leveraging cells generated by a mask-aware variational autoencoder-generative adversarial network (VAE-GAN), reducing oversmoothing of imputed values and preserving biologically meaningful heterogeneity. Across benchmarks, scASprofiler enhances delineation of cell populations and recovery of splicing signals. When applied to datasets generated using plate- and droplet-based platforms, scASprofiler uncovers cryptic AS events and reveals cell-type-specific AS patterns that complement and extend insights derived from gene expression. Together, our study establishes scASprofiler as a robust and versatile tool for dissecting AS landscapes from scRNA-seq data.

## Introduction

Alternative splicing (AS) [1] critically expands transcriptomic and proteomic diversity by reshaping exon-intron architectures, thereby influencing cell identity, developmental trajectories, and disease states [2–5]. Single-cell RNA sequencing (scRNA-seq) enables the characterization of AS at cellular resolution, revealing splicing dynamics across individual cells [6–11]. However, junction-level signals in scRNA-seq data remain markedly sparse, particularly in droplet-based platforms with inherent 3′-end bias [12, 13]. The limitations, stemming from shallow sequencing depth and protocol-dependent biases, further aggravate junction dropout, constraining the power to resolve cell populations and detect cell-type-specific splicing.

Several computational methods for AS analysis from scRNA-seq data have been developed, each addressing distinct challenges in splicing detection and quantification [14–25]. SpliZ [26] introduces an annotation-free splicing *Z* score that identifies regulated splicing events directly from splice-junction evidence. scQuint [27] quantifies intron usage based on junction read counts and enables robust differential splicing across cell types and tissues. BRIE2 [16] employs a Bayesian regression framework to relate event-level splicing proportions to cell-level features, incorporating uncertainty propagation. Methods such as Outrigger [21] construct splice graphs to define events and characterize splicing modalities. SCATS [28] targets low-coverage settings for differential AS analysis. SCASL [25] proposes a splicing-centric representation to investigate cellular heterogeneity even under sparse coverage. SCSES [24] presents a network diffusion-based imputation method designed to accurately recover splicing changes across major types of splicing events at the single-cell level. Collectively, these methods advance splicing event definition, quantification, and differential splicing analysis at single-cell resolution, refining AS detection and interpretation in complex datasets. Despite these advances, missing data remain pervasive in the splice-junction read count matrix. SCASL relies on a neighbor-based imputation strategy, which may over-smooth splicing signals and obscure biologically meaningful variation. Furthermore, the imputation performance of SCSES is severely limited for rare cell populations due to the imbalance.

Here, we introduce scASprofiler, a tailored deep convolutional generative network designed to restore missing junction-level splicing values at the single-cell resolution, thereby enhancing the accuracy and sensitivity of single-cell AS analysis. scASprofiler employs a mask-aware strategy to generate virtual cells that retain authentic splicing signals based on a variational autoencoder-generative adversarial network (VAE-GAN) [29–31] framework, and these virtual profiles are subsequently used to impute missing junction read counts. scASprofiler is inspired by GAN-generated-data imputation strategies, which estimate missing values by first synthesizing realistic complete samples and then using these synthetic profiles to guide imputation [32]. Across diverse single-cell datasets, scASprofiler demonstrates significantly superior performance compared to other algorithms and can be effectively applied to cell clustering. Moreover, the junction-level estimates produced by scASprofiler can be used to uncover additional cell-type-specific AS patterns that complement gene expression. scASprofiler can be applied to analyze AS in scRNA-seq data generated from both plate- and droplet-based platforms. Together, scASprofiler as a highly promising and versatile tool for single-cell splicing analysis is established.

## Materials and methods

### Data preprocessing

scRNA-seq datasets generated by the SMART-seq [33] and 10× Genomics [34] platforms were obtained from the National Center for Biotechnology Information (NCBI) Gene Expression Omnibus (GEO) [35] and European Nucleotide Archive (ENA) [36]. Distinct preprocessing pipelines were applied to plate-based Smart-seq2 data and droplet-based 10× Genomics data.

For Smart-seq2 RNA-seq data, raw reads were trimmed to remove adapters using Trim Galore (v0.6.10) [37]. Trimmed reads were aligned to the reference genomes (human *hg38* or mouse *mm10*) using STAR (v2.7.10b) [38]. Splice-junction read counts were extracted from SJ.out.tab files generated in one-pass alignment mode. Gene expression quantification was performed using RSEM (v1.3.3) [39] on BAM files produced by STAR in two-pass mode.

For the 10× Genomics dataset, raw reads were aligned to the default GRCh38 using Cell Ranger (v9.0.1) [34] to generate cell barcodes and BAM files. Reads with valid barcodes were retained using the subset-bam utility provided by 10× Genomics, and bamtools (v2.5.2) [40] was used to split the filtered BAM file into single-cell BAM files. Each single-cell BAM file was then realigned to the reference genome using STAR with default parameters, and splice-junction read counts files were generated.

Following the strategy implemented in scQuint, AS events sharing the same 5′ or 3′ splice site were grouped into AS modules for Smart-seq2 datasets. For droplet-based 10× Genomics data, inspired by MARVEL, splice junctions were aggregated at the gene level, with all junctions assigned to the same gene being treated as a single gene-centric AS module.

To mitigate spurious signals arising from low sequencing depth, AS modules with ultralow coverage (<20 total junction reads across all AS sites within a module) were discarded. Junction counts from all retained AS sites and cells were assembled into a splice-junction read counts matrix, where rows correspond to AS sites and columns correspond to individual cells. Quality filtering was applied to retain AS sites observed in ˃10 cells and cells with read counts in ˃1000 AS sites. The resulting filtered splice-junction read counts matrix was denoted as ${\mathrm{X}}_{\mathrm{pre}}\in{\mathrm{R}}^{N\times K}$, where *N* is the number of retained AS sites and *K* is the number of retained cells.

### Conversion of splicing information into images

scASprofiler normalizes the splice-junction read counts matrix on a per-cell basis by dividing each cell’s counts by its maximum junction count, scaling values to the interval [0,1]. To enable self-supervised training, a predefined fraction of observed entries (10% by default) is randomly masked to simulate missingness, yielding a random matrix. Each splice-junction vector is padded to the smallest square dimension *n × n* satisfying *n*^2^ ≥ *N* and reshaped column-wise into a 2D grayscale image. Correspondingly, a binary random mask matrix that distinguishes observed from missing entries is generated and reshaped into a grayscale image. Cell labels *y* for conditional training were derived from the corresponding gene-expression profiles of the same cells. When curated cell-type annotations were available for a dataset (e.g. provided by the original study or accompanying metadata), we directly used these annotations as *y*. Otherwise, we generated pseudo-labels by unsupervised clustering of the gene-expression matrix using Scanpy: gene expression counts were library-size normalized and log-transformed, highly variable genes were selected, principal component analysis (PCA) [41] was performed for dimensionality reduction, a KNN graph was constructed in the PCA space, and communities were identified using the Leiden [42] algorithm. The resulting cluster assignments were used as pseudo-labels *y* and one-hot encoded for label conditioning in the VAE-GAN. For a dataset containing K cells, this procedure produces K paired data-mask images, which are used to train a VAE-GAN-based model that reconstructs missing junction signals while preserving observed values.

### Model architecture of scASprofiler

The core of scASprofiler is a mask-aware, label-conditioned VAE-GAN that jointly learns a latent representation of splice-junction read counts, while explicitly accounting for dropout-induced structured sparsity via the mask. The encoder projects splicing images into a continuous latent space, the decoder reconstructs or generates class-conditioned splicing images from latent space, and the discriminator operates on paired data-mask inputs to provide both a learned denoising signal and an adversarial training objective. These three components are trained end to end, enabling the model to capture high-dimensional junction-usage patterns consistent with the observed data, the cell labels, and the structured sparsity introduced by dropout.

The encoder is a lightweight convolutional network that progressively compresses the input image into a compact feature representation and outputs the mean and log-variance $\left(\mu, \log{\sigma}^2\right)$ of a Gaussian posterior ${q}_{\phi}\left(z\mid x\right)$. The latent space is sampled using the standard re-parameterization trick, $z=\mu +\sigma \odot \epsilon$, with $\epsilon \sim \mathcal{N}\left(0,I\right)$. To improve robustness across varying input resolutions, the hidden dimensionality of the encoder’s intermediate fully connected layer is determined automatically from the flattened convolutional features using a fixed scaling rule.

The decoder reconstructs a splicing image $\hat{x\ }$from *z*while being explicitly conditioned on class labels. Specifically, *z* is projected to a spatial feature map, and the one-hot class label is spatially expanded and convolutionally transformed to match the feature-map resolution, and the resulting feature maps are concatenated along the channel dimension. The fused representation is then mapped through convolutional layers to produce a single-channel reconstruction, with outputs constrained to [0,1] via a sigmoid activation. In addition to reconstruction from encoder-sampled *z*, the decoder can generate label-conditioned samples by drawing $z\sim \mathcal{N}\left(0,I\right)$.

The discriminator takes the concatenated data-mask pair $\left[x;m\right]$and is also conditioned on the one-hot class label. It extracts a joint representation of the input and label, and outputs an image-shaped denoising map that is up-sampled to the original resolution *H× W*.

During training, the discriminator is applied to three input types using shared parameters: (a) real data–mask pairs, (b) VAE reconstructions $\left[{\hat{x}}_{\mathrm{rec}};m\right]$, and (c) prior-generated samples $\left[{\hat{x}}_{\mathrm{gen}};m\right]$. This adversarial component complements the variational reconstruction objective and encourages realistic, class-consistent splice-junction signal patterns under dropout.

### Training objective and optimization

scASprofiler is trained under an equilibrium generative adversarial framework using three coupled objectives for encoder, decoder, and discriminator. All image-space reconstruction, adversarial, discriminator, and feature-reconstruction losses are computed in a mask-aware manner so that only observed positions contribute, whereas the KL term regularizes the latent posterior.

The decoder is optimized to minimize a combination of reconstruction and adversarial terms. For reconstructions, the discriminator is applied to real inputs and to inputs reconstructed by the VAE branch. A reconstruction loss is defined as the masked absolute difference between the discriminator outputs for the real image *x* and its reconstructed image ${\hat{x}}_{\mathrm{rec}}$:


$${\mathcal{L}}_{\mathrm{rec}}=\frac{\Sigma \mid D\left(x\oplus m\right)-D\left({\hat{x}}_{\mathrm{rec}}\oplus m\right)\mid \odot m}{\sum m}$$

where *D* denotes the discriminator, *⊕* denotes concatenation of the data and mask channels, and the sums are run over all pixels.

For the adversarial alignment, the discriminator outputs on random generations and VAE reconstructions are compared to the corresponding decoder outputs with masked absolute error. Two masked ${\ell}_1$ terms are computed, one for random generations and one for VAE reconstructions, and averaged. The decoder loss is given by a weighted sum of the reconstruction loss and the average adversarial ${\ell}_1$ loss, with a relatively small weight on the reconstruction term to stabilize training. Gradients from this loss update only the decoder parameters. Specifically, we define the two masked ${\ell}_1$ terms as:


\begin{eqnarray*}&& {L}_{\mathrm{fake}}=\frac{\sum \mid D\left({\hat{x}}_{gen}\oplus m\right)-{\hat{x}}_{gen}\mid \odot m}{\sum m} \\&& {L}_{\mathrm{vae}} =\frac{\sum \mid D\left({\hat{x}}_{rec}\oplus m\right)-{\hat{x}}_{rec}\mid \odot m}{\sum m.} \end{eqnarray*}

Their average forms the adversarial alignment loss:


$${L}_{\mathrm{adv}}=\frac{1}{2}\left({L}_{\mathrm{fake}}+{L}_{\mathrm{vae}}\right)$$

The decoder objective is then


$${L}_{\mathrm{dec}}=\lambda{L}_{\mathrm{rec}}+{L}_{\mathrm{adv}},\lambda \ll 1$$

The discriminator is trained to distinguish real inputs from both random generations and VAE reconstructions while maintaining an equilibrium between reconstruction quality and adversarial discrimination. Let ${L}_{\mathrm{real}}$ denote the masked absolute difference between discriminator output and the real image. We define


$${L}_{\mathrm{real}}=\frac{\sum \left|D\left(x\oplus m\right)-x\right|\odot m}{\sum m}$$

The discriminator loss is defined as


$${\displaystyle \begin{array}{c}{\mathcal{L}}_D={\mathcal{L}}_{\mathrm{real}}-k\frac{{\mathcal{L}}_{\mathrm{fake}}+{\mathcal{L}}_{\mathrm{vae}}}{2}\end{array}}$$

where *k* is a scalar control variable that balances the relative weight of real and fake terms. After each optimization step, *k* is updated according to


$${\displaystyle \begin{array}{c}k\leftarrow \mathrm{clip}\left(k+{\lambda}_k\;\mathrm{diff},0,1\right)\end{array}}$$

with


$${\displaystyle \begin{array}{c}\mathrm{diff}=\gamma\;{\mathcal{L}}_{\mathrm{real}}-\frac{{\mathcal{L}}_{\mathrm{fake}}+{\mathcal{L}}_{\mathrm{vae}}}{2}\end{array}}$$

where *γ* and ${\lambda}_k$ are fixed hyperparameters. This update encourages the system to maintain a desired equilibrium between the reconstruction loss on real samples and the average loss on fake samples.

The encoder is optimized with a combination of KL divergence and discriminator feature reconstruction. For a batch of inputs, the approximate posterior parameters *μ* and $\log{\sigma}^2$ produced by the encoder are regularized toward a standard normal prior with a KL term


$${\displaystyle \begin{array}{c}{\mathcal{L}}_{\mathrm{KL}}=-\frac{1}{2}\frac{1}{D_z}\sum \left(1+\log{\sigma}^2-{\mu}^2-{\sigma}^2\right)\end{array}}$$

where the sum runs over all latent dimensions and ${D}_z$ denotes the latent dimensionality. In addition, discriminator outputs on VAE reconstructions and real inputs are compared using masked squared error to define a feature reconstruction loss


$${\displaystyle \begin{array}{c}{\mathcal{L}}_{\mathrm{feat}}=\frac{\sum{\left(D\left({\hat{x}}_{\mathrm{rec}}\oplus m\right)-D\left(x\oplus m\right)\right)}^2\odot m}{\sum m}\end{array}}$$

The encoder loss is then given by


$${\displaystyle \begin{array}{c}{\mathcal{L}}_E={\mathcal{L}}_{\mathrm{KL}}+\alpha\;{\mathcal{L}}_{\mathrm{feat}}\end{array}}$$

where *α* is a fixed weight that controls the relative contribution of feature matching. Gradients from this loss update only the encoder parameters.

Encoder, decoder, and discriminator are optimized alternately on each batch using Adam optimizers with a shared learning rate and fixed momentum parameters. The training loop iterates over mini-batches of masked splicing images and labels, and progresses through a predefined maximum number of epochs or until early stopping criteria are met.

### Early stopping based on masked validation error

To prevent overfitting, scASprofiler uses early stopping based on reconstruction error evaluated on a pseudo-validation mask. Before training, we sample a fixed matrix of latent codes ${Z}_{\mathrm{fixed}}$ from a standard normal distribution (one latent vector per cell) and pair these codes with one-hot labels derived from the original class assignments; both are kept fixed during training. At the end of each epoch, the decoder maps ${Z}_{\mathrm{fixed}}$ and the labels to reconstructed splicing images, which are reshaped to the original feature-by-cell layout to form a dense surrogate matrix. We then compute the mean squared error between this matrix and the raw data restricted to pseudo-validation positions, defined as entries that were truly observed but artificially masked. If this validation error decreases by more than a predefined minimum relative to the best value observed so far, the current encoder, decoder, and discriminator parameters are saved as the best model and the patience counter is reset; if no such improvement occurs for a preset number of successive epochs, training is terminated and the best model is used for downstream imputation.

### Imputation of missing data values

After training, scASprofiler treats the learned decoder as a class-conditioned generative prior and couples it with a KNN strategy to fill in missing splice-junction values. For each cell cluster, *s* latent vectors are sampled from a standard normal distribution and decoded together with the corresponding one-hot label to generate virtual splicing profiles. For a given cell, Euclidean distances between its masked feature vector and the virtual profiles are computed only over observed entries to identify the k-nearest virtual neighbors. The default *k* was set to 10 to balance noise reduction and preservation of cell-level splicing heterogeneity. A smaller *k* preserves local similarity but may be more sensitive to noise, whereas an overly large *k* may over-smooth cell-specific splicing patterns. The robustness of this default setting was further evaluated by *k*-sensitivity analysis (Supplementary Fig. S23) across multiple datasets. For each AS site *j*, a KNN-based estimate ${\tilde{c}}_{i,j}^{\left(k\mathrm{NN}\right)}$ is obtained. The final imputed matrix ${\hat{c}}_{i,j}$ is then defined elementwise as


$${\displaystyle \begin{array}{c}{\hat{c}}_{i,j}=\left\{\begin{array}{cc}{c}_{i,j},& \mathrm{if}\ {c}_{i,j}\ge 0\\{}{\tilde{c}}_{i,j}^{\left(k\mathrm{NN}\right)},& \mathrm{else}\end{array}\right.\end{array}}$$

### Quantification of alternative splicing events

Inspired by scQuint [27], we grouped AS events that share the same 5′ or 3′ splice site into an AS module in plate-based Smart-seq2 datasets. For each cell *i* and AS site *j* belonging to module *m*, ${c}_{i,j}$ denotes the splice-junction read count. The AS probability is then defined as


$${\displaystyle \begin{array}{c}{p}_{i,j}=\frac{c_{i,j}}{\sum_{j^{\prime}\in m}{c}_{i,{j}^{\prime }}}\end{array}}$$

Because droplet-based 10× Genomics data exhibited strong 3′-end bias and sparse junction coverage, a shared donor/acceptor splice-site definition would discard many informative junctions. Therefore, inspired by MARVEL [23], AS sites were aggregated at the gene level. All AS sites arising from the same gene *g* were treated as a gene-centric AS module *G(g)**.* For a cell *i* and junction *j* belonging to gene $\mathrm{g}$, the junction-level splicing proportion was computed as:


$${\displaystyle \begin{array}{c}{p}_{i,j}=\frac{c_{i,j}}{\sum_{j^{\prime}\in G(g)}{c}_{i,{j}^{\prime }}}\end{array}}$$

### scASprofiler differential alternative splicing analysis

For plate-based Smart-seq2 datasets, we assessed differential AS on the imputed data using scQuint, specifically with the run_differential_splicing function from the scquint.differential_splicing module. For droplet-based 10× Genomics datasets, we used MARVEL to test for differential splicing on AS probabilities. Pairwise comparisons between cell types were carried out with the CompareValues function, using the Anderson–Darling (“ad”) method as the test statistic and MARVEL’s default multiple-testing correction.

### Functional enrichment analysis

We conducted functional enrichment analysis on the identified differentially spliced genes to provide initial insights into the functional changes associated with each cluster and key developmental stages. The clusterProfiler package (v4.10.1) [43] was used for GO pathway enrichment analysis, while the org.Hs.eg.db (v3.18.0) and org.Mm.eg.db packages (v3.18.0) [44] were used for Entrez ID conversion.

## Results

### Overview of scASprofiler

scASprofiler is a deep convolutional generative network designed to accurately recover splice-junction read counts from scRNA-seq data. Each cell’s splicing profile is converted into a grayscale image that serves as the model’s input. During model inference, scASprofiler also produces an imputed splice-junction read counts matrix, from which AS probabilities are subsequently calculated for downstream analyses.

**Figure 1:**
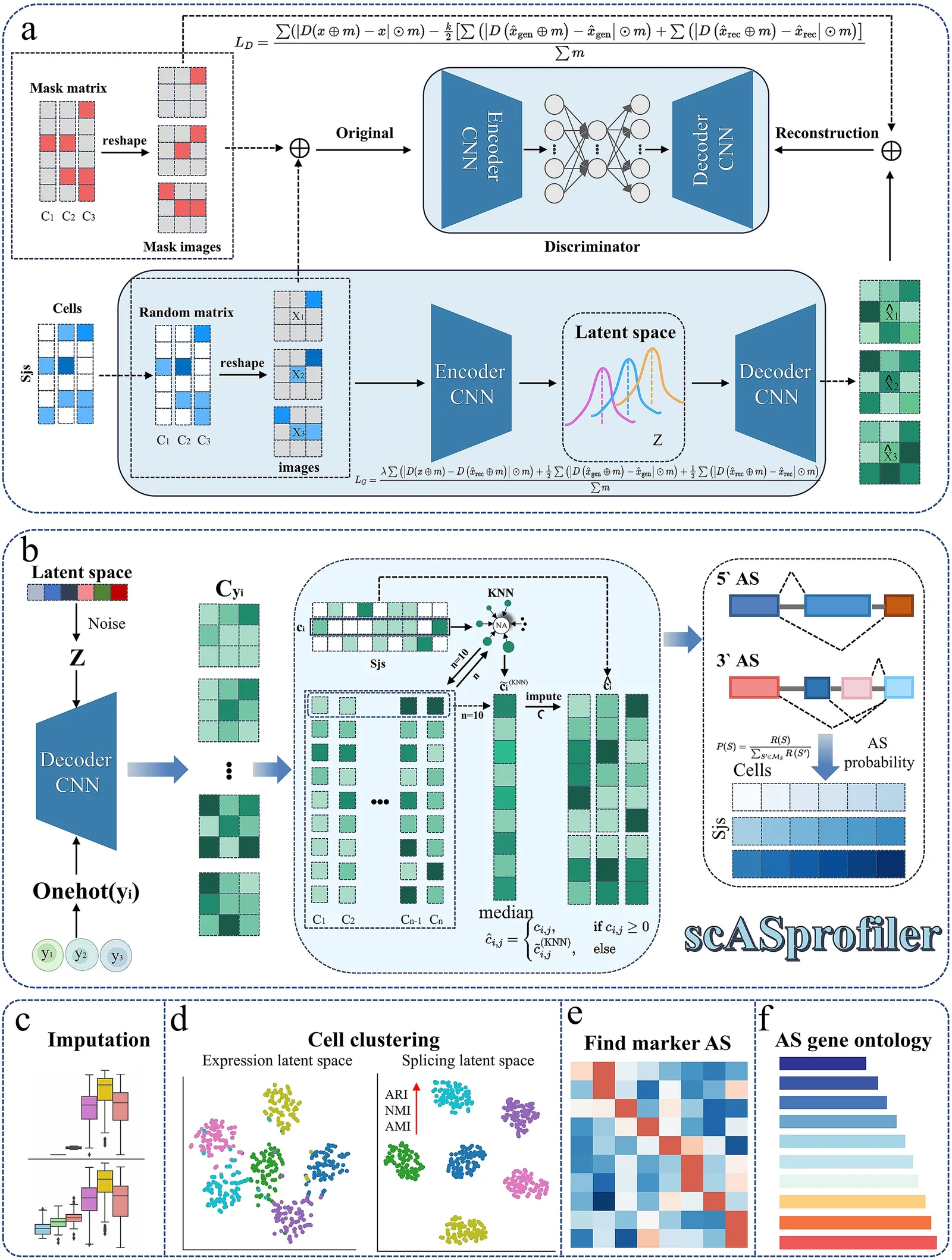
scASprofiler workflow for single-cell AS analysis; (a) the splice-junction read counts matrix is reshaped into image-like cell profiles, where grayscale intensities encode junction usage and a paired binary mask matrix denotes observed versus missing entries; a convolutional encoder–decoder trained under a VAE–GAN framework learns a latent distribution while reconstructing inputs; the discriminator constrains the model to generate biologically plausible reconstruction that preserves cell-specific splicing patterns; (b) the decoder generates class-conditioned virtual splicing profiles by sampling a noise vector *z* and one-hot cell labels; for each query cell, KNN are identified based solely on observed splice-junction positions; missing splice-junction read counts are imputed by aggregating junction signals across the neighbors; right: schematic illustrating event-level quantification derived from the imputed splice-junction read counts matrix; (c) distributional fidelity and reconstruction performance are benchmarked on data before and after imputation; (d) low-dimensional embeddings constructed from the imputed data reveal compact, label-concordant clusters; clustering metrics (ARI, NMI, AMI) are reported; (e) differential splicing analysis identifies cell-type-associated AS events; (f) the functional enrichment analysis of marker AS events.

The method comprises two major components. First, scASprofiler ingests the raw splice-junction read counts matrix and randomly masks a predefined fraction of observed values (non-missing) to generate a self-supervised training random matrix. This masking strategy allows the model to assess its capacity to reconstruct true splicing signals and provides an early-stopping criterion. The random matrix is reshaped into a grayscale image, while a binary mask matrix, constructed from the random matrix to delineate observed and missing values, is encoded as a grayscale image. The paired images serve as inputs to a VAE-GAN framework [29–31], which is trained until the predefined stopping criteria are met (Fig. 1a). Second, the trained generative model is used to generate a predetermined number of virtual cells of each cell cluster. The generated cells provide reference splice-junction values to guide the imputation of missing values in the raw dataset. For each cell, the K-Nearest Neighbors (KNN) [45] method is applied over the observed junction positions to identify similar virtual cells, and the median of the similar cells is used to fill in the missing values of the raw data (Fig. 1b). After imputation, AS probabilities are computed by grouping splice-junction counts according to splice-junction structure. The imputed splice-junction read counts matrix produced by scASprofiler enables a broad range of downstream analyses (Fig. 1c–f), including imputation accuracy evaluation, splicing-based cell clustering, identification of marker AS events, and functional enrichment of AS-associated genes. Together, scASprofiler yields refined and biologically coherent splicing representations for downstream single-cell analyses.

We further evaluated the computational behavior of scASprofiler across datasets with different numbers of cells and splice-junction features. The model showed stable convergence within 13–52 early-stopping epochs, while runtime and peak memory usage increased with input scale. These results support the practical applicability of scASprofiler to both Smart-seq2 and 10× scRNA-seq datasets (Supplementary Fig. S1 and Supplementary Table S1).

### scASprofiler robustly imputes splice-junction read counts across diverse datasets

To rigorously evaluate the imputation performance of scASprofiler, we benchmarked the model across diverse scRNA-seq datasets [21, 46, 47] spanning multiple tissues and biological contexts, while systematically simulating increasing dropout frequencies. These datasets included three full-length collections: induced pluripotent stem cells (iPSCs) and endoderm cells (PRJEB15062) [21], a time-course differentiation series from H1 embryonic stem cells (ESC) to definitive endoderm cells (DEC) (GSE75748) [46], and a lineage progression dataset comprising iPSCs, neural progenitor cells (NPCs), and motor neurons (MNs) (GSE85908) [47]. Performance was assessed using four complementary criteria: (i) comparative evaluation against existing methods using multiple clustering metrics; (ii) distributional consistency between originally measured and imputed junction counts at artificially masked positions; (iii) quantitative divergence between the distributions of originally measured and imputed junction counts at artificially masked positions; and (iv) t-SNE [48] derived from raw and imputed data. Across all analyses, scASprofiler exhibited strong and resilient performance under increasing dropout frequencies.

**Figure 2:**
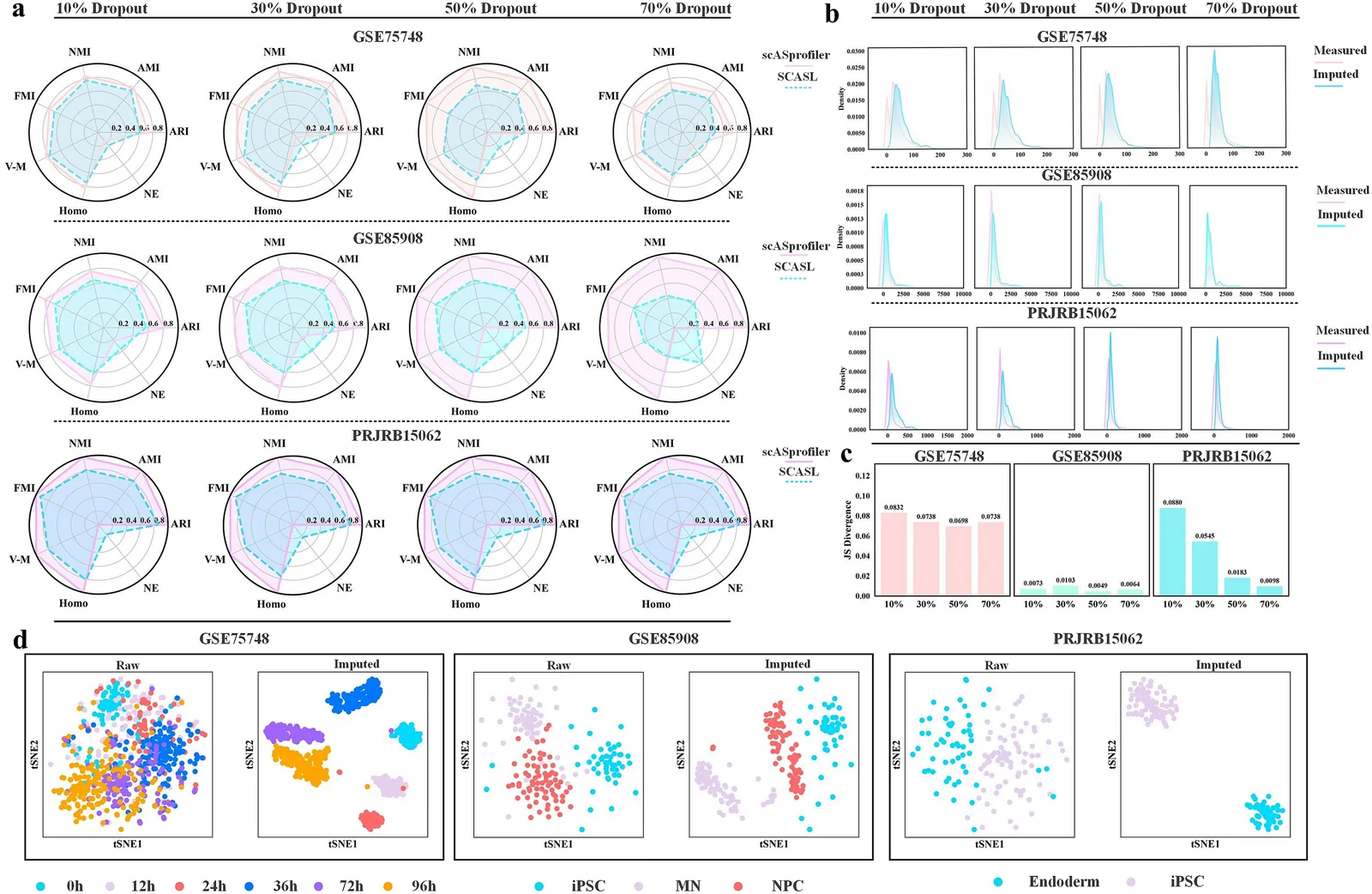
Imputation performance of scASprofiler across diverse datasets and dropout frequencies; (a) radar plots comparing scASprofiler and SCASL across increasing dropout frequencies on three datasets (GSE75748, GSE85908, PRJEB15062) using clustering metrics (ARI, AMI, NMI, FMI, Homo, V-M, NE); (b) density curves of measured versus imputed values at artificially masked positions across dropout frequencies; (c) JS divergence between imputed and raw data distributions at artificially masked positions across dropout frequencies; (d) t-SNE embeddings derived from AS probability matrix, comparing raw and imputed data; cells are colored by cell type or time point.

We first compared scASprofiler with SCASL, a recent method for splicing imputation, using a comprehensive panel of clustering metrics: Adjusted Rand Index (ARI), Adjusted Mutual Information (AMI), Normalized Mutual Information (NMI), Fowlkes–Mallows Index (FMI), Homogeneity (Homo), V-measure (V-M), and normalized entropy (NE) [49–53]. Radar plots in Fig. 2a and Supplementary Fig. S3a summarize performance across dropout frequencies and diverse datasets. In dataset GSE75748, scASprofiler sustained high ARI and NMI values across all dropout frequencies. For example, at 50% dropout, ARI and NMI reached 0.98 and 0.97, respectively, whereas SCASL yielded markedly lower scores (ARI: 0.54, NMI: 0.70). Even at 70% dropout, the performance of scASprofiler remained robust, whereas SCASL degraded significantly. Similar robustness was observed in GSE85908, where scASprofiler maintained stable ARI (>0.8) and NMI (>0.7) across all dropout frequencies, compared with substantial deterioration in SCASL. In PRJEB15062, scASprofiler consistently outperformed SCASL across all metrics, particularly in Homo under 60%–70% dropout. These results reveal a clear trend: scASprofiler surpasses SCASL under every dropout condition and dataset, with a widening performance margin as sparsity increases. In addition, clustering visualizations (Supplementary Fig. S2) show that scASprofiler not only preserves inter-cell similarity but also improves resolution of fine-grained cell populations.

To examine distributional fidelity, we compared density curves of raw and imputed data at artificially masked positions across dropout frequencies (Fig. 2b and Supplementary Fig. S3b). In GSE75748, scASprofiler generated imputed distributions that closely mirrored the raw data even at 70% dropout. Similar stability was observed in GSE85908, where imputed distributions maintained appropriate modal structure and dispersion at moderate dropout frequencies. PRJEB15062 showed similarly high fidelity, with reconstructed densities tracking the original distributions even under extreme sparsity. These results indicate that scASprofiler reliably recovers the global splicing landscape with minimal distortion across heterogeneous data conditions.

We next quantified distributional similarity using Jensen–Shannon (JS) divergence [54, 55], computed between imputed and raw values at artificially masked positions. As shown in Fig. 2c and Supplementary Fig. S3c, scASprofiler exhibited consistently low JS divergence across dropout frequencies. In GSE75748, divergence was 0.0832 at 10% dropout and 0.0738 at 70% dropout, indicating only a modest shift despite severe sparsity. GSE85908 displayed even more stable behavior (JS <0.04 across all dropout levels), and PRJEB15062 also showed a low divergence overall (maximum 0.0880). These results underscore the model’s ability to generate distributionally faithful splicing values under progressively worsening missingness.

Two-dimensional t-SNE embeddings derived from imputed data showed improved preservation of biological structure (Fig. 2d). In GSE75748, raw data exhibited a diffuse manifold with extensive mixing between adjacent time points. Imputation markedly sharpened separation, producing compact and well-ordered developmental clusters. In GSE85908, scASprofiler clearly separated distinct cell clusters: MNs and NPCs formed distinct, internally homogeneous clusters, and iPSCs cleanly separated from MNs and NPCs. For PRJEB15062, imputation transformed a diffuse elongated cloud into two well-segregated clusters with minimal overlap. Across diverse datasets, imputed representations consistently displayed tighter within-class cohesion and clearer between-class boundaries. To assess whether the learned splicing representation depended on label conditioning or AS-site ordering, we performed label-control and random AS-site-ordering analyses. In the label-control analysis, no-label, shuffled-label, and splicing-label controls showed that the recovered splicing representation was not solely determined by the conditioning labels (Supplementary Figs S4 and S5). In the random AS-site-ordering analysis, five independent random feature orders produced broadly consistent cell-type or developmental-stage separation, and clustering metrics remained stable across random orderings, suggesting that scASprofiler was not primarily dependent on a specific image-like feature layout (Supplementary Figs S6 and S7). Systematic ablation analyses further showed dataset-dependent performance loss after removing or replacing major components, supporting the contribution of the main design elements of scASprofiler (Supplementary Fig. S8).

Collectively, these results demonstrate that scASprofiler provides a robust, distributionally accurate, and biologically coherent solution for imputing single-cell splicing data across a wide range of dropout frequencies and experimental conditions. Its ability to preserve fine-scale structure and minimize distributional distortion makes scASprofiler a powerful tool for downstream AS analysis in high-throughput single-cell sequencing.

### scASprofiler significantly improves visualization across diverse single-cell contexts

We next assessed the generalizability of scASprofiler across diverse single-cell settings by benchmarking its visualization and clustering performance. Across all scenarios, scASprofiler consistently produced markedly clearer cell-type separations than SCASL, SCSES, scQuint, BRIE2, and SpliZ.

**Figure 3:**
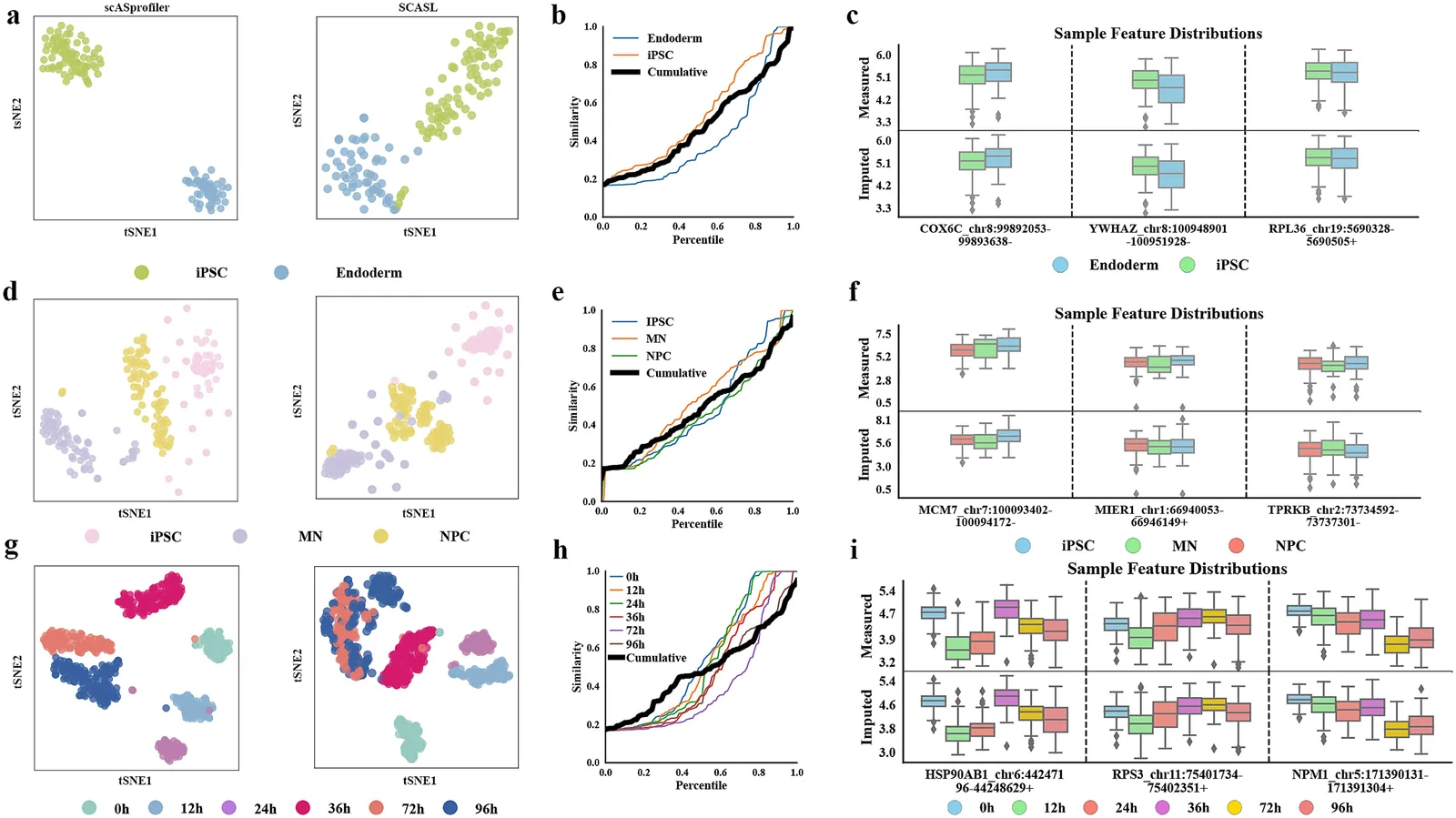
Benchmarking scASprofiler across scRNA-seq datasets; (a) t-SNE embeddings of iPSC and endoderm cells using imputed splice-junction features generated by scASprofiler (left) and SCASL (right); (b) cumulative distribution of similarity between measured and imputed values across the iPSC and endoderm cells; (c) boxplots of three representative splice events showing the agreement between measured and imputed distributions in iPSC and endoderm; (d) t-SNE embeddings of iPSC, MN, and NPC cells for scASprofiler (left) and SCASL (right); (e) cumulative similarity distributions between measured and imputed values across iPSC, MN, and NPC; (f) boxplots of three representative splice events illustrating concordance between measured and imputed distributions in iPSC, MN, and NPC; (g) t-SNE embeddings of differentiation time-course cells (0, 12, 24, 36, 72, 96 h) for scASprofiler (left) and SCASL (right); (h) cumulative similarity distributions between measured and imputed values across time points; (i) boxplots of three representative splice events demonstrating agreement between measured and imputed distributions across the six differentiation stages.

In iPSC and endoderm cells (Fig. 3a), scASprofiler resolved two compact, non-overlapping clusters, whereas SCASL yielded a diffuse manifold with extensive intermixing. The imputed values are consistent with measured values, exhibiting low distributional divergence [mean JS distance = 0.5172, standard deviation (SD) = 0.2814; Fig. 3b]. The three splice events with the highest similarity between measured and imputed values are shown in Fig. 3c (mean JS distances = 0.022, 0.030, and 0.018), demonstrating faithful preservation of splicing changes across different cell types. In datasets comprising NPC, MN, and iPSC, scASprofiler again produced three well-separated clusters concordant with reference annotations (Fig. 3d), whereas SCASL exhibited substantial cross-type mixing. Distributional concordance between imputed and measured values remained high (mean JS distance = 0.5146, SD = 0.2664; Fig. 3e). Representative high-similarity splice events (mean JS distances = 0.278, 0.250, and 0.148; Fig. 3f) further confirmed preservation of biologically meaningful splicing differences. Along the ESC-to-DEC differentiation trajectory, cells sampled across six time points were projected by SCASL into a diffuse, elongated manifold, whereas scASprofiler reorganized them into six discrete, temporally ordered clusters (Fig. 3g). The imputed values closely recapitulated the distribution of measured values across stages (mean JS distance = 0.5134, SD = 0.3082; Fig. 3h). Three representative splice events with high concordance (mean JS distances = 0.085, 0.076, and 0.108; Fig. 3i) further demonstrate robust preservation of stage-specific splicing dynamics. Given that existing single-cell splicing tools differ substantially in their design goals and output formats, we further evaluated scASprofiler together with representative methods, including SCSES, scQuint, BRIE2, and SpliZ, using a unified downstream assessment based on t-SNE visualization and clustering concordance. Across PRJEB15062, GSE85908, and GSE75748, scASprofiler produced a more compact and annotation-consistent t-SNE space. scQuint, BRIE2, and SpliZ showed varying degrees of cell-type mixing or reduced separation of developmental stages. SCSES showed clear separation in PRJEB15062, but was not available for GSE85908 and did not consistently match the performance of scASprofiler in the remaining evaluated datasets (Supplementary Fig. S9). Quantitative evaluation using ARI, AMI, NMI, FMI, V-M, Homo, and NE further supported the visual results, with scASprofiler showing the most favorable overall clustering performance across the benchmark datasets (Supplementary Fig. S10). We also compared scASprofiler with two scRNA-seq imputation methods, SSNMDI [56] and scRNMF [57], which were originally developed for gene-expression matrix imputation rather than splice-junction-level AS profiling. Although these methods improved some representations on small datasets, they showed less consistent recovery of splicing-defined cell structures, particularly in the mouse brain dataset, where scASprofiler preserved clearer cell-type-specific organization and showed better clustering concordance (Supplementary Fig. S11).

Collectively, these results demonstrate that scASprofiler delivers robust, high-resolution visualization across diverse cellular systems, capturing discrete populations with enhanced sensitivity and preserving biologically meaningful splicing variation.

### scASprofiler substantially enhances alternative splicing detection during induced pluripotent stem cell-to-endoderm differentiation

**Figure 4:**
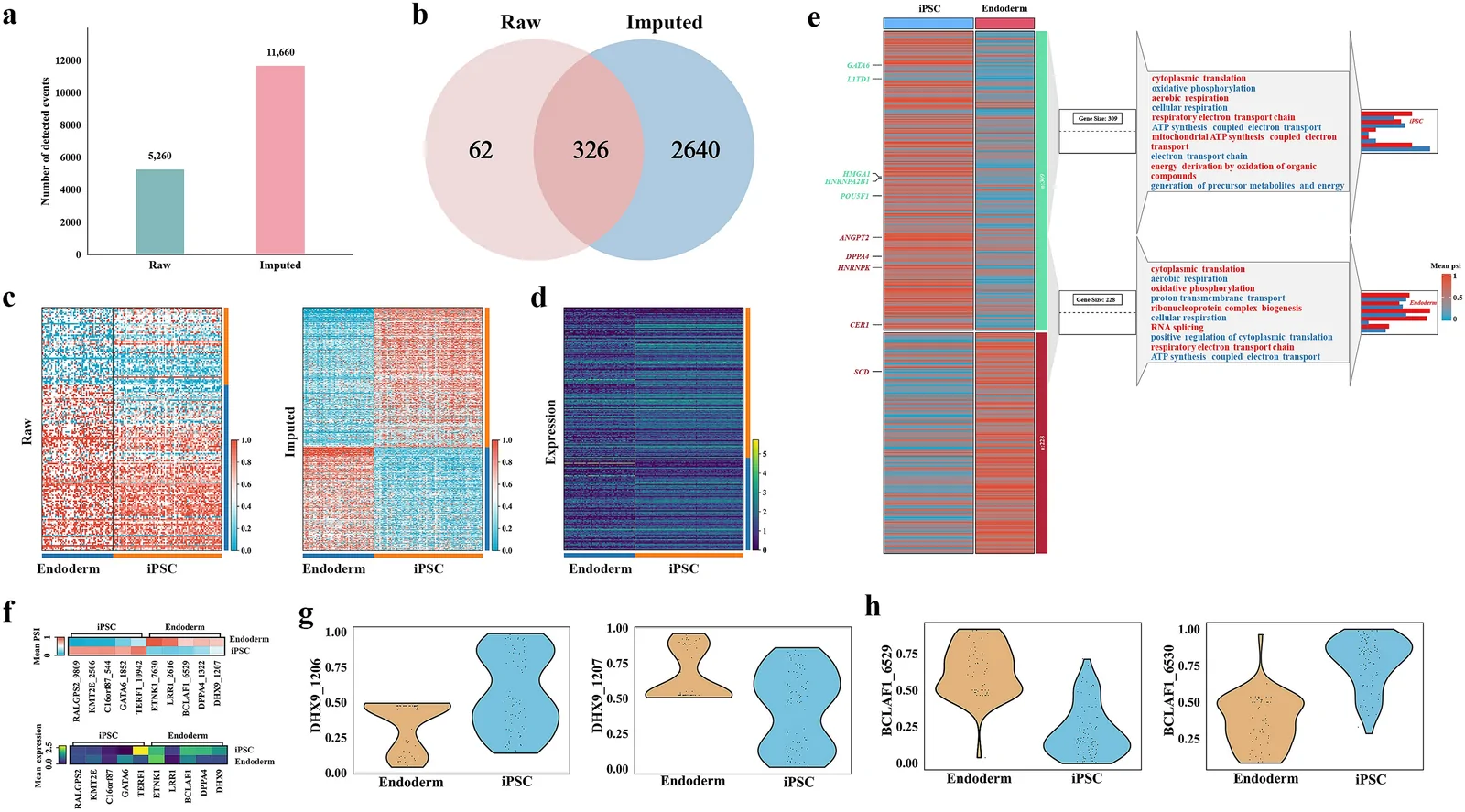
AS detection during iPSC-to-endoderm differentiation by scASprofiler; (a) number of splicing events detected by scASprofiler in raw versus imputed data; (b) overlap of differential AS events identified from raw and imputed data; (c) heatmaps of differential AS events from raw and imputed data (rows: AS events; columns: cells; cells ordered by cell type); (d) gene-expression heatmap of genes associated with the differential AS events; (e) GO analysis of genes corresponding to marker differential AS events, grouped by AS-probabilities-defined modules; (f) comparison of mean AS probabilities of marker AS events with mean expression of their corresponding genes; (g) AS-probability distribution of AS events in DHX9; (h) AS-probability distribution of AS events in BCLAF1.

To evaluate the ability of scASprofiler to resolve AS dynamics at single-cell resolution, we analyzed scRNA-seq data from iPSCs and iPSC-derived endoderm cells. Splicing events were quantified on the raw and imputed data. Imputation markedly increased detection sensitivity: 11 660 splicing events were recovered after imputation, compared with 5260 events identified from raw data under identical parameters (Fig. 4a). Differential splicing analysis further revealed a substantial gain in regulatory signal, with 2966 differentially spliced events detected in the imputed data versus 388 in the raw data, of which 326 were shared (Fig. 4b). These improvements highlight the extent to which sparsity in raw junction counts obscures biologically meaningful AS variation.

The differential AS events from the raw and imputed data were visualized (Fig. 4c). The heatmaps further revealed that the imputed data exhibited distinct clustering patterns, sharply contrasting with the raw data. Specifically, imputation led to a more pronounced clustering of cell types, suggesting that missing or incomplete AS information in the raw data may have masked these biological patterns. Furthermore, we found that the corresponding genes showed no significant differential expression at the gene level (Fig. 4d). This underscores scASprofiler’s ability to uncover AS regulatory changes that are critical yet frequently missed by traditional gene-centric analyses.

To explore the biological relevance of these newly recovered AS events, we performed functional enrichment analysis (Fig. 4e). Two gene modules defined by AS probability (Module I and Module II; 537 genes in total) exhibited strong cellular specificity. Module I, enriched in iPSC, was associated with cytoplasmic translation, ribosome biogenesis and protein synthesis, reflecting enhanced biosynthetic and RNA-processing demands in pluripotent cells. Key genes included POU5F1 (OCT4), L1TD1, HMGA1, and HNRNPK [58–61]. Module II, enriched in endoderm, highlighted oxidative phosphorylation and electron-transport chain activity, consistent with metabolic remodeling during germ-layer specification. Representative genes included CER1, SCD, DPPA4 [62–64], and members of the ANGPTL [65] family.

Differential splicing analysis pinpointed numerous marker AS events that robustly distinguished iPSC from endoderm despite no significant changes in gene-level expression (Fig. 4f and Supplementary Fig. S13c). In contrast, markers derived from raw data exhibited weaker effect sizes (Supplementary Fig. S13b), and their corresponding genes also showed no differences in expression between iPSC and endoderm cells (Supplementary Fig. S13d). In addition, several biologically informative splicing events were recovered after imputation (Fig. 4g and h and Supplementary Figs S12 and S13a). For example, two DHX9 [66] splice junctions (DHX9_1206 and DHX9_1207) displayed complementary, state-aligned AS shifts (Fig. 4g), indicating splicing regulation that would be obscured by gene-level summaries. Similarly, two BCLAF1 [67] splice junctions (BCLAF1_6529 and BCLAF1_6530), which were too sparse to be reliably detected in the raw data, emerged only after imputation and showed strong cell-type-specific divergence (Fig. 4h). These AS events fall within the differential alternative splicing (DAS) modules highlighted above, suggesting that splicing regulation is embedded within broader translational and mitochondrial programs associated with definitive endoderm specification.

Collectively, these results demonstrate that scASprofiler substantially enhances the sensitivity, resolution, and interpretability of single-cell AS analysis, enabling the discovery of cell-type-specific AS programs that remain invisible in conventional gene-centric workflows. This method provides a powerful framework for dissecting post-transcriptional regulation during cell fate transitions.

### Constructing cell-type-resolved splicing landscapes in the mouse primary motor cortex

**Figure 5:**
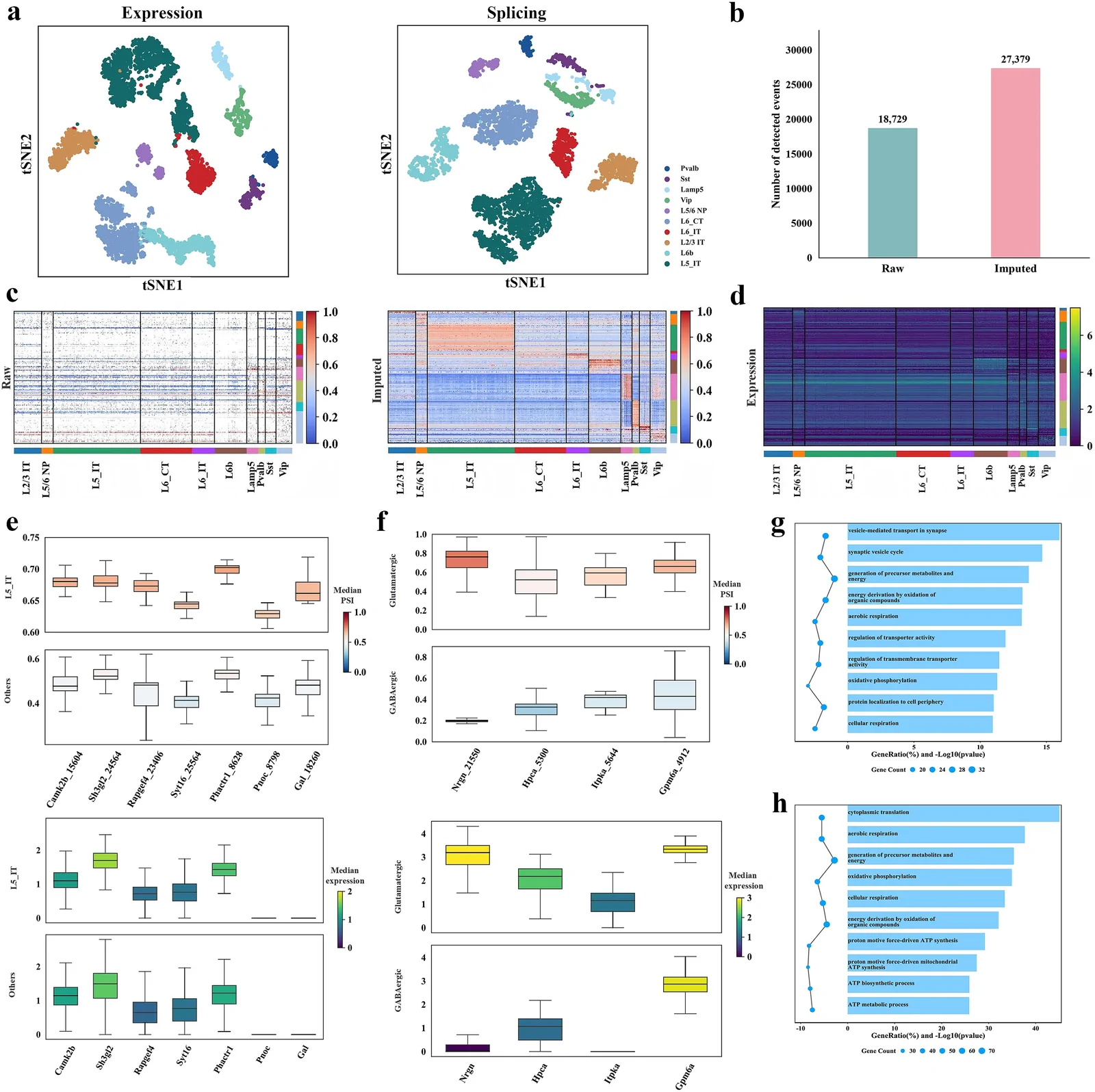
Cell-type-specific splicing patterns in mouse primary motor cortex; (a) t-SNE visualization of gene-expression and splicing latent spaces; (b) number of splicing events detected in raw versus imputed data; (c) heatmaps of AS probability from raw (left) and imputed (right) data; (d) gene expression heatmap for genes corresponding to differential AS events (imputed data), showing weaker separation at the gene level; (e) examples from L5_IT neurons: AS probabilities of marker AS events (top) and corresponding gene expression (bottom); (f) examples comparing inhibitory and excitatory neurons: class-associated AS probabilities (top) and corresponding gene expression (bottom); (g) GO analysis for DAS genes in L5_IT versus other cell types; (h) GO analysis for DAS genes in inhibitory versus excitatory contrasts.

We applied scASprofiler to single-cell data from the mouse primary motor cortex [68] and first compared the splicing latent space to the gene-expression latent space (Fig. 5a). Both latent spaces achieved clear separation of major cell types, indicating that scASprofiler captures biologically meaningful splicing variation.

As a result, we detected 27 379 splicing events by scASprofiler, which is substantially more than the 18 729 events identified from the raw data using identical parameters (Fig. 5b). The biological relevance of these cell-type-specific events is highlighted in Fig. 5c and d and Supplementary Figs S14 and S15. Heatmaps of differential AS events (Fig. 5c) show that imputed data exhibit markedly sharper, cell-type-specific clustering than raw data, suggesting that dropout in the raw data masks underlying splicing structure. Notably, for differential AS events identified by scASprofiler, their corresponding genes show no significant differential expression, indicating that these splicing changes are invisible to conventional gene-level analyses. The gene-expression heatmap in Fig. 5d illustrates this discrepancy, demonstrating that stable transcript abundance can coexist with substantial isoform-level rewiring [69, 70]. Comparison of Supplementary Fig. S14a and b provides specific examples in which AS events discriminate cell types while the corresponding gene expression does not; in contrast, Supplementary Fig. S15a shows that analyses based solely on raw data fail to reveal these patterns. In addition, Supplementary Fig. S15b and c further show that scASprofiler can reveal differentially spliced events obscured in raw data.

In L5_IT neurons, a subset of the top-50 L5_IT-associated splice sites displays distinctly elevated AS probabilities within L5_IT but low usage in other cell types (Fig. 5e, top). Their corresponding gene-expression levels, however, could hardly distinguish this cell type from others (Fig. 5e, bottom). These splice sites arise from genes central to synaptic plasticity and activity-dependent signaling (Camk2b and Rapgef4) [71, 72], synaptic endocytosis and membrane remodeling (Sh3gl2) [73], intracellular membrane trafficking and regulated secretion (Syt16) [74], actin cytoskeleton regulation and neuronal morphology (Phactr1), and neuropeptide-mediated neuromodulation (Pnoc and Gal) [75, 76]. In these loci, AS-probability variation provides finer resolution of L5_IT identity than gene expression, consistent with splicing specialization in intratelencephalic projection neurons. A similar pattern extends across neuronal classes. Among inhibitory (GABAergic) and excitatory (glutamatergic) neurons [77], class-associated splice junctions exhibit sharply segregated AS-probability distribution (Fig. 5f, top), whereas gene-expression summaries are less discriminative (Fig. 5f, bottom). Inhibitory (GABAergic) neurons show low usage of class-associated junctions in Nrgn, Hpca, Itpka, and Gpm6a [78–81], consistent with cell-class-dependent splicing regulation in genes related to postsynaptic Ca2+/calmodulin-associated signaling (Nrgn, Hpca), inositol phosphate (IP3)-associated Ca2+ signaling and spine actin dynamics (Itpka), and neuronal membrane dynamics/neurite outgrowth (Gpm6a) at these splice sites. Excitatory-enriched AS events involve Nrgn, Hpca, and Itpka, consistent with postsynaptic Ca2+/calmodulin-linked signaling, synaptic plasticity, and dendritic integration, together with Gpm6a, consistent with neuronal membrane dynamics and neurite outgrowth. Additional class-distinct events appear in mitochondrial respiratory-chain and ATP synthase genes, though effect sizes vary across splice sites. These junction-level differences align with functional enrichment patterns. In L5_IT versus other types, enriched terms relate to synaptic signaling, vesicle trafficking, and neurotransmitter release (Fig. 5g). In inhibitory versus excitatory comparisons, enrichment terms emphasize energy metabolism, oxidative phosphorylation, and ATP biosynthesis (Fig. 5h). Gene Ontology (GO) enrichment for other cell types is provided in Supplementary Fig. S16.

Collectively, these analyses demonstrate that scASprofiler robustly reconstructs cell-type-specific splicing landscapes in the mouse primary motor cortex, revealing splicing regulation that is largely uncoupled from gene-level expression and inaccessible to conventional pipelines.

### Application of scASprofiler to 10× Genomics single-cell RNA sequencing data

**Figure 6:**
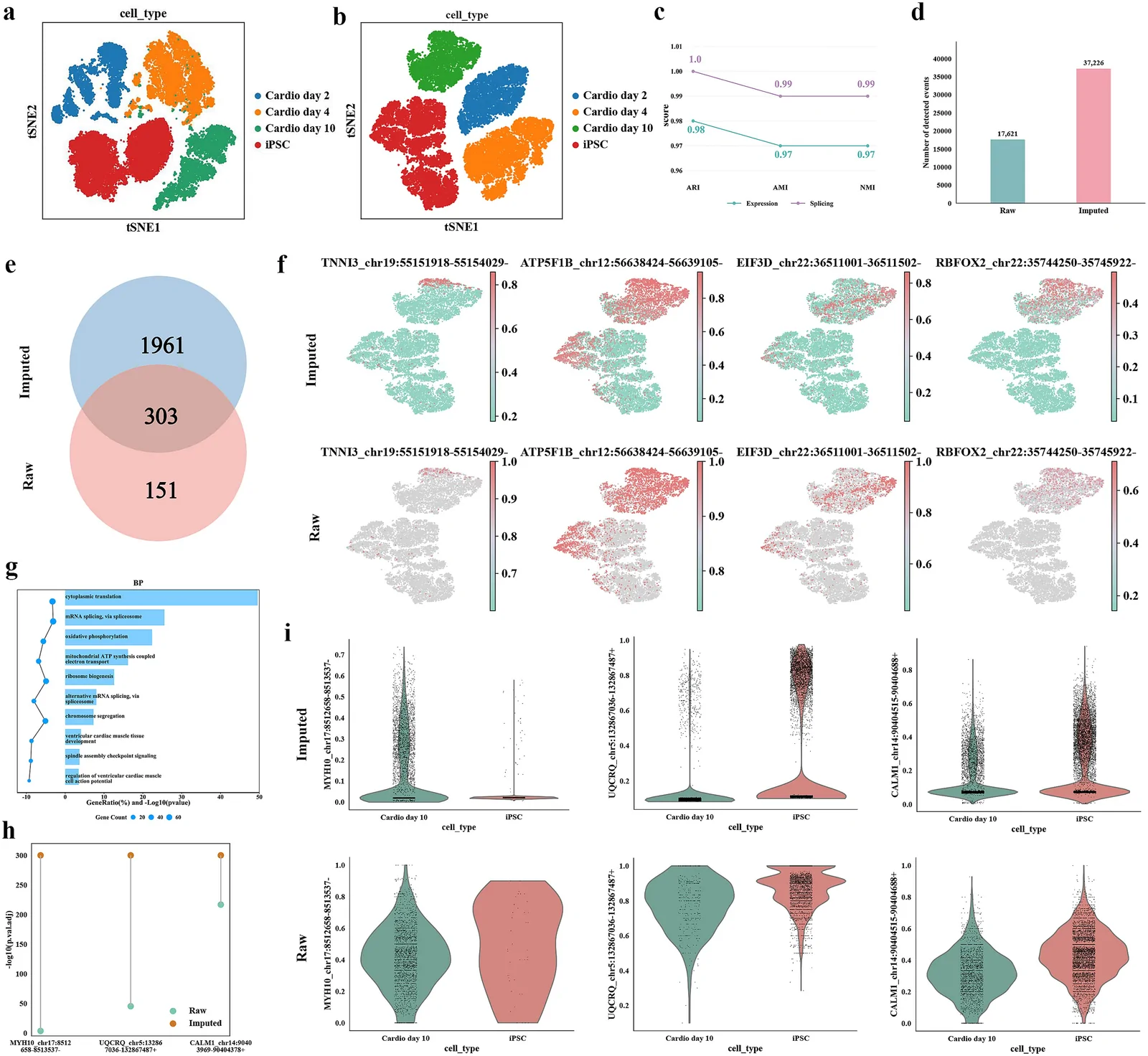
scASprofiler enables robust splicing analysis in droplet-based (10×) scRNA-seq during iPSC-to-cardiomyocyte differentiation; (a) t-SNE embedding of the expression latent space; (b) t-SNE embedding in the splicing latent space; (c) clustering agreement metrics (ARI, AMI, NMI) between splicing- and expression-based latent spaces; (d) number of detectable splice junctions before and after imputation; (e) overlap of DSJs identified in raw versus imputed data for iPSC and Day-10 cardiomyocytes; (f) representative DSJs in TNNI3, ATP5F1B, EIF3D, and RBFOX2 projected onto the embeddings, showing junction usage patterns in imputed (top) and raw (bottom) data; (g) GO enrichment of genes associated with DSJs detected in the imputed data; (h) statistical comparison of shared DSJs from raw and imputed analyses, with imputed data yielding consistently lower *P*-values; (i) violin plots of AS probabilities for MARVEL-shared DSJs in iPSCs and Day-10 cardiomyocytes, contrasting imputed (top) with raw (bottom) estimates.

scASprofiler also enables robust single-cell AS analysis for droplet-based scRNA-seq platforms such as 10× Genomics. To demonstrate its utility, we analyzed a 10× scRNA-seq dataset profiling the differentiation of iPSCs into Day-10 cardiomyocytes [82]. We first compared the splicing latent space with the expression latent space (Fig. 6a and b). Both embeddings recovered the expected cell populations, indicating that junction usage after imputation retains stage-discriminative information comparable to that captured at the expression level. Across multiple clustering metrics (ARI, NMI, and AMI), the splicing latent space exhibited consistently higher concordance with stage labels than the expression latent space, reflecting superior clustering performance (Fig. 6c). Imputation markedly increased the number of detectable splice junctions from 17 621 (raw) to 37 226 (imputed), substantially expanding the analyzable search space (Fig. 6d). Focusing on the iPSCs versus the Day-10 cardiomyocytes, the set of differential splice junctions (DSJs) broadened considerably after imputation: 1961 imputed-only DSJs, 303 shared DSJs, and 151 raw-only DSJs (Fig. 6e). This asymmetry highlights the gain in detectability under sparse coverage, while the shared DSJs demonstrate concordance with signals present in the raw data.

Representative examples illustrate how imputation improves biological separation during differentiation stages (Fig. 6f). A splice junction in TNNI3 [83] is detectable in both raw and imputed data. However, only the imputed data reveal a coherent gradient in AS probability from iPSC to Day-10 cardiomyocytes, whereas the raw data are too sparse to delineate this transition. Junctions in ATP5F1B [84], EIF3D [85], and RBFOX2 [86], detectable only after imputation, show clear state-specific differences in junction usage. These genes map directly onto key axes of cardiomyocyte maturation. TNNI3 encodes cardiac troponin I, whose developmental replacement of fetal isoforms enhances adult-like Ca2+ sensitivity and contractile kinetics. ATP5F1B, the β subunit of mitochondrial ATP synthase, reflects the metabolic transition from glycolysis to oxidative phosphorylation. EIF3D supports translational reprogramming as sarcomeric and mitochondrial proteins accumulate. RBFOX2, a key splicing regulator, remodels cardiac isoforms essential for excitation–contraction coupling and cytoskeletal organization. Together, these examples illustrate how scASprofiler uncovers contractile, metabolic, translational, and post-transcriptional programs associated with cardiomyocyte maturation.

We next compared gene-level changes with junction-level changes. VIM [87], a cytoarchitecture regulator and marker of muscle regeneration, displayed coordinated gene-expression and junction-usage patterns (Supplementary Fig. S17). By contrast, TPM1 [88], encoding a conserved actin-binding tropomyosin critical for cardiac contractility, showed a more complex relationship: overall gene expression increased in cardiomyocytes, yet distinct TPM1 junctions changed in opposite directions. Such decoupling underscores the importance of isoform-resolved analysis beyond gene-level summaries.

GO analysis of DSJ-associated genes from the imputed data revealed strong enrichment for pathways related to translation, mitochondrial protein targeting, oxidative phosphorylation, and respiratory-chain assembly (Fig. 6g), consistent with metabolic and proteostatic reprogramming during differentiation. We further compared DSJs identified by scASprofiler with those detected directly from the raw data (Fig. 6h). For DSJs detectable in both inputs, the imputed data yielded lower *P*-values, reflecting enhanced statistical power and the limitations of conventional raw-matrix approaches.

As an additional consistency check, MARVEL identified splice junctions classified as differential in both inputs (Fig. 6i). For these junctions, AS-probability distributions from the imputed data showed tighter clustering and clearer group separation, indicating that the imputation strategy of scASprofiler sharpens genuine biological signals rather than introducing artifacts.

## Discussion

In this study, we introduce scASprofiler, a generative, splice-junction-centric framework that substantially improves recovery of missing junction signals at single-cell resolution, thereby enhancing the reliability of downstream visualization, clustering, and differential splicing analyses. Across multiple datasets and controlled missingness conditions, scASprofiler consistently outperforms existing methods, demonstrating superior reconstruction fidelity and markedly improved separability of cell types. Even under elevated dropout levels, scASprofiler maintains higher clustering concordance (ARI, NMI, AMI, Homo, V-M, NE) while inducing smaller distortions to junction-usage distributions, as reflected by lower JS divergence. Consequently, the low-dimensional geometry of the splicing space becomes more coherent, and cell identities are more readily resolved on the basis of junction-level features.

These gains arise from several core design principles. First, scASprofiler normalizes junction counts and reformulates each cell’s junction profile into an image-like representation, enabling mask-aware generative reconstruction that explicitly incorporates a binary missingness mask for each matrix entry [89–91]. Second, to avoid information leakage and over-smoothing, cell–cell similarity is computed solely from observed entries, and missing values are filled using KNN aggregation based on median neighbor profiles. This strategy preserves biologically meaningful variability while mitigating sparsity-driven distortions. In addition, scASprofiler incorporates several safeguards to reduce overfitting by decoder-generated virtual cells. These include self-supervised masking of observed junction values, held-out early stopping based on reconstruction error, mask-aware loss computation, Kullback–Leibler (KL)-based latent-space regularization, and observed-position-based KNN median imputation. Importantly, virtual cells do not directly overwrite entire single-cell splicing profiles; rather, they serve as reference profiles for local reconstruction, with each real cell matched to virtual cells only through its originally observed junction positions. These design choices reduce the likelihood that the model memorizes the training matrix.

To further evaluate whether imputed splicing signals reflect biologically supported variation rather than overfitting or label-conditioning artifacts, we performed a series of downstream robustness analyses on the PRJEB15062 iPSC-to-endoderm dataset. First, label-control analyses showed that the default label-conditioned results were substantially recapitulated under no-label, shuffled-label, and splicing-derived-label settings. These control settings recovered a large shared core of DAS events with the default analysis, with consistent effect sizes, significance rankings, and top-ranked event overlap (Supplementary Fig. S18). This result indicates that the main DAS signals were not solely determined by the conditioning labels, although biological label conditioning increased the sensitivity for detecting additional cell-state-associated events. Second, pseudo-bulk validation using LeafCutter [92] (Supplementary Fig. S19) provided independent support for imputed DAS events: 80.5% of imputed DAS introns showed exact intron-coordinate support, and 80.0% of DAS groups were supported at the pseudo-bulk level, with a median exact-matched intron fraction of 1.0 across DAS groups. Third, permutation-based negative controls (Supplementary Fig. S20) showed that the number and significance of DAS events detected under true labels were clearly separated from the permuted-label background, supporting that the observed DAS signal was not expected under random label assignment. Fourth, random-seed analyses (Supplementary Fig. S21) demonstrated stable DAS detection across independent model training runs, with comparable DAS counts, high pairwise Jaccard similarity, recurrent DAS events across seeds, consistent effect sizes, and stable directionality. Finally, random AS-site-ordering analyses (Supplementary Fig. S22) showed substantial overlap and correlation between default-order and random-order results, indicating that the downstream DAS signals were not driven by a specific feature ordering in the image-like representation. Although these validation analyses were performed on PRJEB15062 rather than across all datasets, they collectively support the conclusion that scASprofiler enhances reproducible, biologically supported splicing signals rather than generating arbitrary virtual-cell-driven patterns.

The strengthened signal enables substantially deeper biological discovery. During iPSC-to-endoderm differentiation, scASprofiler increases detectable splicing events from 5260 to 11 660 and boosts differential splicing events from 388 to 2966, revealing splicing regulation that is largely invisible at the gene-expression level yet sharply demarcates developmental states. In the mouse primary motor cortex, imputation expands the event space from 18 729 to 27 379 and enhances the resolution of splicing signatures distinguishing inhibitory and excitatory neurons as well as specific subpopulations such as L5_IT. In the iPSC-to-cardiomyocyte dataset generated by a droplet-based platform, detectable junctions increase from 17 621 to 37 226, with 1961 DSJs emerging only after imputation. Because the downstream robustness analyses and independent pseudo-bulk LeafCutter validation were primarily performed on PRJEB15062, future studies using matched pseudo-bulk, long-read, or deeply sequenced reference data across additional datasets will be valuable for further validating imputation-derived splicing events in broader cellular contexts.

Sequence regulation is a fundamental determinant of splice-site recognition and AS. Although sequence-derived information, such as splice-site strength, flanking sequence motifs, RNA-binding protein motifs, or embeddings from pretrained sequence models, could provide useful priors for imputing missing splice-junction values, scASprofiler was designed in this study as a junction-count-driven framework to alleviate the sparsity of splice-junction read count matrices in scRNA-seq data. We did not incorporate sequence-derived features at this stage because these features describe intrinsic properties of splice sites or local sequence context, whereas the current model focuses on imputing missing junction values from observed single-cell junction-count patterns, missingness masks, and cell-level conditioning information.

Although this study focuses on the development of computational methods, representative splicing events identified by scASprofiler should be experimentally validated. For example, DHX9 and BCLAF1 splice junctions in the PRJEB15062 iPSC-to-endoderm system could be detected in independent iPSC and iPSC-derived endoderm samples. Junction-specific primers could be used to quantify the relative abundance of the selected splice junctions, while primers flanking the alternatively spliced region could be used to distinguish the corresponding isoforms. These events could be examined by RT-PCR [93], RT-qPCR [94], or targeted amplicon sequencing [95], and PCR products could be further confirmed by Sanger sequencing [96].

Overall, scASprofiler offers a principled and scalable approach to junction-centric single-cell splicing analysis. By recovering biologically meaningful splicing signals under realistic coverage limitations, refining splicing-based cellular structure, and revealing regulatory programs complementary to gene-expression analyses, scASprofiler provides higher-resolution insight into cell states and facilitates the discovery of cell-type-specific splicing programs that remain inaccessible to conventional pipelines.

## Conclusion

We present scASprofiler, a junction-centric framework for AS analysis in scRNA-seq data that combines mask-aware generative modeling with neighborhood-based reconstruction to robustly recover sparse splice-junction signals across plate- and droplet-based platforms. Across diverse datasets, scASprofiler improves clustering concordance, preserves junction-usage distributions under dropout, and markedly increases the number of detectable and differentially spliced events. In applications ranging from iPSC-to-endoderm differentiation and mouse motor cortex to 10× Genomics cardiomyocyte maturation, scASprofiler reveals cell type- and state-specific splicing programs that are largely invisible at the gene-expression level, providing an accurate and scalable complement to conventional gene-centric workflows.

**Key Points**

- scASprofiler addresses the extreme sparsity of splice-junction read counts that limits alternative splicing (AS) analysis in single-cell RNA sequencing (scRNA-seq) data.
- scASprofiler uses a tailored deep convolutional generative network with a mask-aware variational autoencoder-generative adversarial network strategy to impute missing junction read counts.
- The method reduces over-smoothing of imputed values and preserves biologically meaningful splicing heterogeneity at single-cell resolution.
- Across benchmark datasets, scASprofiler improves cell population delineation and enhances the recovery of informative splicing signals.
- Applications to plate- and droplet-based scRNA-seq datasets reveal cryptic and cell-type-specific AS patterns that complement gene-expression analyses.

## Supplementary Material

Supplementary_File_bbag497_corrected

## Data Availability

All single-cell data utilized in this manuscript were sourced from publicly available databases and were deposited in the GEO (GSE75748, GSE85908, GSE130731) and ENA (PRJEB15062). The BICCN Cortex data were downloaded from https://assets.nemoarchive.org/dat-ch1nqb7.

## Code availability

Codes for all the data analyses in this study are available in GitHub (https://github.com/1027victor/scASprofiler).

## Declarations

Ethics approval and consent to participate are not applicable to this study.

## References

[1] Marasco LE, Kornblihtt AR. The physiology of alternative splicing. *Nat Rev Mol Cell Biol* 2023;24:242–54. 10.1038/s41580-022-00545-z 36229538

[2] Cortés-López M, Chamely P, Hawkins AG et al. Single-cell multi-omics defines the cell-type-specific impact of splicing aberrations in human hematopoietic clonal outgrowths. Cell Stem Cell 2023;30:1262–81.e8. 10.1016/j.stem.2023.07.012 PMC10528176 37582363

[3] Lu Y, Lin K, Ruan Y et al. Deciphering alternative splicing patterns during cell fate transition of fast chemical reprogramming. *BMC Biol* 2025;23:164. 10.1186/s12915-025-02264-1 40490773 PMC12150459

[4] Rogalska ME, Vivori C, Valcárcel J. Regulation of pre-mRNA splicing: roles in physiology and disease, and therapeutic prospects. *Nat Rev Genet* 2023;24:251–69. 10.1038/s41576-022-00556-8 36526860

[5] Tao Y, Zhang Q, Wang H et al. Alternative splicing and related RNA binding proteins in human health and disease. *Signal Transduct Target Ther* 2024;9:26. 10.1038/s41392-024-01734-2 38302461 PMC10835012

[6] Yang X, Tong Y, Liu G et al. scAPAatlas: an atlas of alternative polyadenylation across cell types in human and mouse. *Nucleic Acids Res* 2022;50:D356–64. 10.1093/nar/gkab917 34643729 PMC8728290

[7] Xing Y, Yang W, Liu G et al. Dynamic alternative splicing during mouse preimplantation embryo development. *Front Bioeng Biotechnol* 2020;8:35. 10.3389/fbioe.2020.00035 32117919 PMC7019016

[8] Chi H, Zhang Y, Li A et al. Analysis of alternative splicing heterogeneity during early stages of mouse embryonic development. Current Bioinformatics 2026;21:330–43. 10.2174/0115748936376211250429102627

[9] Gaiti F, Hawkins A, Chamely P et al. Single-cell multi-omics defines the cell-type specific impact of SF3B1 splicing factor mutations on hematopoietic differentiation in human clonal hematopoiesis and myelodysplastic syndromes. *Blood* 2021;138:145. 10.1182/blood-2021-147529

[10] Tian C, Zhang Y, Tong Y et al. Single-cell RNA sequencing of peripheral blood links cell-type-specific regulation of splicing to autoimmune and inflammatory diseases. *Nat Genet* 2024;56:2739–52. 10.1038/s41588-024-02019-8 39627432 PMC11631754

[11] Wang J, Wen S, Chen M et al. Regulation of endocrine cell alternative splicing revealed by single-cell RNA sequencing in type 2 diabetes pathogenesis. *Commun Biol* 2024;7:778. 10.1038/s42003-024-06475-0 38937540 PMC11211498

[12] Woo H, Eyun S-I. Applications and techniques of single-cell RNA sequencing across diverse species. *Brief Bioinform* 2025;26:bbaf354. 10.1093/bib/bbaf354 40698863 PMC12284766

[13] Luo H, Hussain A, Abbas M et al. Droplet-based single-cell RNA sequencing: decoding cellular heterogeneity for breakthroughs in cancer, reproduction, and beyond. *J Transl Med* 2025;23:1091. 10.1186/s12967-025-06996-0 41088302 PMC12522672

[14] Dehghannasiri R, Olivieri JE, Damljanovic A et al. Specific splice junction detection in single cells with SICILIAN. *Genome Biol* 2021;22:219. 10.1186/s13059-021-02434-8 34353340 PMC8339681

[15] Huang Y, Sanguinetti G. BRIE: transcriptome-wide splicing quantification in single cells. *Genome Biol* 2017;18:123. 10.1186/s13059-017-1248-5 28655331 PMC5488362

[16] Huang Y, Sanguinetti G. BRIE2: computational identification of splicing phenotypes from single-cell transcriptomic experiments. *Genome Biol* 2021;22:251. 10.1186/s13059-021-02461-5 34452629 PMC8393734

[17] Liu S, Chen X, Huang X et al. AEnet: a practical tool to construct the splicing-associated phenotype atlas at a single cell level. *Gigascience* 2025;14:giaf110. 10.1093/gigascience/giaf110 40990037 PMC12457822

[18] Najar CFBA, Burra P, Yosef N et al. Identifying cell state–associated alternative splicing events and their coregulation. *Genome Res* 2022;32:1385–97. 10.1101/gr.276109.121 35858747 PMC9341514

[19] Olivieri JE, Dehghannasiri R, Wang PL et al. RNA splicing programs define tissue compartments and cell types at single-cell resolution. *Elife* 2021;10:e70692. 10.7554/eLife.70692 34515025 PMC8563012

[20] Song K, Zheng Y, Zhao B et al. DOLPHIN advances single-cell transcriptomics beyond gene level by leveraging exon and junction reads. *Nat Commun* 2025;16:6202. 10.1038/s41467-025-61580-w 40615408 PMC12227669

[21] Song Y, Botvinnik OB, Lovci MT et al. Single-cell alternative splicing analysis with Expedition reveals splicing dynamics during neuron differentiation. Mol Cell 2017;67:148–61.e5. 10.1016/j.molcel.2017.06.003 PMC5540791 28673540

[22] Van Hecke M, Beerenwinkel N, Lootens T et al. ELLIPSIS: robust quantification of splicing in scRNA-seq. *Bioinformatics* 2025;41:btaf028. 10.1093/bioinformatics/btaf028 39936571 PMC11878791

[23] Wen WX, Mead AJ, Thongjuea S. MARVEL: an integrated alternative splicing analysis platform for single-cell RNA sequencing data. *Nucleic Acids Res* 2023;51:e29. 10.1093/nar/gkac1260 36631981 PMC10018366

[24] Wen X, Lv X, Guo D et al. Deciphering splicing heterogeneity at single-cell resolution by SCSES. *Nat Commun* 2025;16:9459. 10.1038/s41467-025-64517-5 41145486 PMC12559242

[25] Xiang X, He Y, Zhang Z et al. Interrogations of single-cell RNA splicing landscapes with SCASL define new cell identities with physiological relevance. *Nat Commun* 2024;15:2164. 10.1038/s41467-024-46480-9 38461306 PMC10925056

[26] Olivieri JE, Dehghannasiri R, Salzman J. The SpliZ generalizes ‘percent spliced in’to reveal regulated splicing at single-cell resolution. *Nat Methods* 2022;19:307–10. 10.1038/s41592-022-01400-x 35241832 PMC9089759

[27] Benegas G, Fischer J, Song YS. Robust and annotation-free analysis of alternative splicing across diverse cell types in mice. *Elife* 2022;11:e73520. 10.7554/eLife.73520 35229721 PMC8975553

[28] Hu Y, Wang K, Li M. Detecting differential alternative splicing events in scRNA-seq with or without unique molecular identifiers. *PLoS Comput Biol* 2020;16:e1007925. 10.1371/journal.pcbi.1007925 32502143 PMC7299405

[29] Goodfellow IJ, Pouget-Abadie J, Mirza M et al. Generative adversarial nets. *Adv Neural Inf Process Syst* 2014;27:2672–80.

[30] Kingma DP, Welling M . Auto-encoding variational Bayes. 2nd International Conference on Learning Representations (ICLR 2014). Banff, AB, Canada: International Conference on Learning Representations (ICLR), 2014, 1–14. 10.48550/arXiv.1312.6114

[31] Larsen ABL, Sønderby SK, Larochelle H et al. Autoencoding beyond pixels using a learned similarity metric. In: Balcan MF, Weinberger KQ (eds.), Proceedings of the 33rd International Conference on Machine Learning, vol. 48. New York, NY, USA: PMLR, 2016, 1558–66.

[32] Hammad Alharbi H, Kimura M . Missing data imputation using data generated by GAN. In: Proceedings of the 2020 3rd International Conference on Computing and Big Data (ICCBD ’20). New York, NY, USA: Association for Computing Machinery, 2020, 73–77. 10.1145/3418688.3418701

[33] Ramsköld D, Luo S, Wang Y-C et al. Full-length mRNA-seq from single-cell levels of RNA and individual circulating tumor cells. *Nat Biotechnol* 2012;30:777–82. 10.1038/nbt.2282 22820318 PMC3467340

[34] Zheng GX, Terry JM, Belgrader P et al. Massively parallel digital transcriptional profiling of single cells. *Nat Commun* 2017;8:14049. 28091601 10.1038/ncomms14049 PMC5241818

[35] Edgar R, Domrachev M, Lash AE. Gene expression omnibus: NCBI gene expression and hybridization array data repository. *Nucleic Acids Res* 2002;30:207–10. 10.1093/nar/30.1.207 11752295 PMC99122

[36] Leinonen R, Akhtar R, Birney E et al. The European nucleotide archive. *Nucleic Acids Res* 2010;39:D28–31. 10.1093/nar/gkq967 20972220 PMC3013801

[37] Krueger F . Trim Galore!: A Wrapper around Cutadapt and FastQC to Consistently Apply Adapter and Quality Trimming to FastQ Files, with Extra Functionality for RRBS Data. Cambridge, UK: Babraham Institute, 2015.

[38] Dobin A, Davis CA, Schlesinger F et al. STAR: ultrafast universal RNA-seq aligner. *Bioinformatics* 2013;29:15–21. 10.1093/bioinformatics/bts635 23104886 PMC3530905

[39] Li B, Dewey CN. RSEM: accurate transcript quantification from RNA-seq data with or without a reference genome. *BMC Bioinformatics* 2011;12:323. 10.1186/1471-2105-12-323 21816040 PMC3163565

[40] Barnett DW, Garrison EK, Quinlan AR et al. BamTools: a C++ API and toolkit for analyzing and managing BAM files. *Bioinformatics* 2011;27:1691–2. 10.1093/bioinformatics/btr174 21493652 PMC3106182

[41] Maćkiewicz A, Ratajczak W. Principal components analysis (PCA). *Comput Geosci* 1993;19:303–42. 10.1016/0098-3004(93)90090-R

[42] Traag VA, Waltman L, van Eck NJ . From Louvain to Leiden: guaranteeing well-connected communities. Sci Rep 2019;9:5233. 10.1038/s41598-019-41695-z PMC6435756 30914743

[43] Yu G, Wang L-G, Han Y et al. clusterProfiler: an R package for comparing biological themes among gene clusters. *OMICS* 2012;16:284–7. 10.1089/omi.2011.0118 22455463 PMC3339379

[44] Gentleman RC, Carey VJ, Bates DM et al. Bioconductor: open software development for computational biology and bioinformatics. *Genome Biol* 2004;5:R80. 10.1186/gb-2004-5-10-r80 15461798 PMC545600

[45] Dudani SA . The distance-weighted k-nearest-neighbor rule. IEEE Trans Syst Man Cybern 1976;SMC-6:325–7. 10.1109/TSMC.1976.5408784

[46] Chu L-F, Leng N, Zhang J et al. Single-cell RNA-seq reveals novel regulators of human embryonic stem cell differentiation to definitive endoderm. *Genome Biol* 2016;17:173. 10.1186/s13059-016-1033-x 27534536 PMC4989499

[47] Linker SM, Urban L, Clark SJ et al. Combined single-cell profiling of expression and DNA methylation reveals splicing regulation and heterogeneity. *Genome Biol* 2019;20:30. 10.1186/s13059-019-1644-0 30744673 PMC6371455

[48] van der Maaten L, Hinton G . Visualizing data using t-SNE. J Mach Learn Res 2008;9:2579–605.

[49] Hubert L, Arabie P. Comparing partitions. *J Classif* 1985;2:193–218. 10.1007/BF01908075

[50] Vinh NX, Epps J, Bailey J. Information theoretic measures for clusterings comparison: variants, properties, normalization and correction for chance. *J Mach Learn Res* 2010;11:2837–54.

[51] Fowlkes EB, Mallows CL. A method for comparing two hierarchical clusterings. *J Am Stat Assoc* 1983;78:553–69. 10.1080/01621459.1983.10478008

[52] Rosenberg A, Hirschberg J . V-measure: a conditional entropy-based external cluster evaluation measure. In: Eisner J (ed.), Proceedings of the 2007 Joint Conference on Empirical Methods in Natural Language Processing and Computational Natural Language Learning (EMNLP-CoNLL). Prague, Czech Republic: Association for Computational Linguistics, 2007, 410–20.

[53] Shannon CE . A mathematical theory of communication. *Bell Syst Tech J* 1948;27:379–423. 10.1002/j.1538-7305.1948.tb01338.x

[54] Endres DM, Schindelin JE. A new metric for probability distributions. *IEEE Trans Inf Theory* 2003;49:1858–60. 10.1109/TIT.2003.813506

[55] Lin J . Divergence measures based on the Shannon entropy. *IEEE Trans Inf Theory* 1991;37:145–151. 10.1109/18.61115

[56] Qiu Y, Yan C, Zhao P et al. SSNMDI: a novel joint learning model of semi-supervised non-negative matrix factorization and data imputation for clustering of single-cell RNA-seq data. *Brief Bioinform* 2023;24:bbad149. 37122068 10.1093/bib/bbad149

[57] Qian Y, Zou Q, Zhao M et al. scRNMF: an imputation method for single-cell RNA-seq data by robust and non-negative matrix factorization. *PLoS Comput Biol* 2024;20:e1012339. 10.1371/journal.pcbi.1012339 39116191 PMC11338450

[58] Nichols J, Zevnik B, Anastassiadis K et al. Formation of pluripotent stem cells in the mammalian embryo depends on the POU transcription factor Oct4. *Cell* 1998;95:379–91. 10.1016/S0092-8674(00)81769-9 9814708

[59] Wong RC-B, Ibrahim A, Fong H et al. L1TD1 is a marker for undifferentiated human embryonic stem cells. *PLoS One* 2011;6:e19355. 10.1371/journal.pone.0019355 21559406 PMC3084827

[60] Shah SN, Kerr C, Cope L et al. HMGA1 reprograms somatic cells into pluripotent stem cells by inducing stem cell transcriptional networks. *PLoS One* 2012;7:e48533. 10.1371/journal.pone.0048533 23166588 PMC3499526

[61] Bakhmet EI, Nazarov IB, Gazizova AR et al. hnRNP-K targets open chromatin in mouse embryonic stem cells in concert with multiple regulators. *Stem Cells* 2019;37:1018–29. 10.1002/stem.3025 31021473

[62] Madan B, Madan V, Weber O et al. The pluripotency-associated gene Dppa4 is dispensable for embryonic stem cell identity and germ cell development but essential for embryogenesis. *Mol Cell Biol* 2009;29:3186–203. 10.1128/MCB.01970-08 19332562 PMC2682008

[63] Iwashita H, Shiraki N, Sakano D et al. Secreted cerberus1 as a marker for quantification of definitive endoderm differentiation of the pluripotent stem cells. *PLoS One* 2013;8:e64291. 10.1371/journal.pone.0064291 23717584 PMC3661443

[64] Ben-David U, Gan Q-F, Golan-Lev T et al. Selective elimination of human pluripotent stem cells by an oleate synthesis inhibitor discovered in a high-throughput screen. *Cell Stem Cell* 2013;12:167–79. 10.1016/j.stem.2012.11.015 23318055

[65] Kirsch N, Chang L-S, Koch S et al. Angiopoietin-like 4 is a Wnt signaling antagonist that promotes LRP6 turnover. Dev Cell 2017;43:71–82.e6. 10.1016/j.devcel.2017.09.011 29017031

[66] Chakraborty P, Huang J, Hiom K. DHX9 helicase promotes R-loop formation in cells with impaired RNA splicing. *Nat Commun* 2018;9:4346. 10.1038/s41467-018-06677-1 30341290 PMC6195550

[67] Savage KI, Gorski JJ, Barros EM et al. Identification of a BRCA1-mRNA splicing complex required for efficient DNA repair and maintenance of genomic stability. *Mol Cell* 2014;54:445–59. 10.1016/j.molcel.2014.03.021 24746700 PMC4017265

[68] Yao Z, Liu H, Xie F et al. A transcriptomic and epigenomic cell atlas of the mouse primary motor cortex. *Nature* 2021;598:103–10. 10.1038/s41586-021-03500-8 34616066 PMC8494649

[69] Dam SH, Olsen LR, Vitting-Seerup K. Expression and splicing mediate distinct biological signals. *BMC Biol* 2023;21:220. 10.1186/s12915-023-01724-w 37858135 PMC10588054

[70] Topa H, Honkela A. Analysis of differential splicing suggests different modes of short-term splicing regulation. *Bioinformatics* 2016;32:i147–55. 10.1093/bioinformatics/btw283 27307611 PMC4908367

[71] O’Leary H, Lasda E, Bayer KU. CaMKIIβ association with the actin cytoskeleton is regulated by alternative splicing. *Mol Biol Cell* 2006;17:4656–65. 10.1091/mbc.e06-03-0252 16928958 PMC1635389

[72] Woolfrey KM, Srivastava DP, Photowala H et al. Epac2 induces synapse remodeling and depression and its disease-associated forms alter spines. *Nat Neurosci* 2009;12:1275–84. 10.1038/nn.2386 19734897 PMC2754861

[73] Watanabe S, Mamer LE, Raychaudhuri S et al. Synaptojanin and endophilin mediate neck formation during ultrafast endocytosis. Neuron 2018;98:1184–97.e6. 10.1016/j.neuron.2018.06.005 PMC6086574 29953872

[74] Cao P, Yang X, Südhof TC. Complexin activates exocytosis of distinct secretory vesicles controlled by different synaptotagmins. *J Neurosci* 2013;33:1714–27. 10.1523/JNEUROSCI.4087-12.2013 23345244 PMC3711587

[75] Zhou X, Stine C, Prada PO et al. Development of a genetically encoded sensor for probing endogenous nociceptin opioid peptide release. *Nat Commun* 2024;15:5353. 10.1038/s41467-024-49712-0 38918403 PMC11199706

[76] Wynick D, Thompson SW, McMahon SB. The role of galanin as a multi-functional neuropeptide in the nervous system. *Curr Opin Pharmacol* 2001;1:73–7. 10.1016/S1471-4892(01)00006-6 11712539

[77] Jin H, Li M, Jeong E et al. A body–brain circuit that regulates body inflammatory responses. *Nature* 2024;630:695–703. 10.1038/s41586-024-07469-y 38692285 PMC11186780

[78] Pak JH, Huang FL, Li J et al. Involvement of neurogranin in the modulation of calcium/calmodulin-dependent protein kinase II, synaptic plasticity, and spatial learning: a study with knockout mice. *Proc Natl Acad Sci U S A* 2000;97:11232–7. 10.1073/pnas.210184697 11016969 PMC17183

[79] Palmer CL, Lim W, Hastie PG et al. Hippocalcin functions as a calcium sensor in hippocampal LTD. *Neuron* 2005;47:487–94. 10.1016/j.neuron.2005.06.014 16102532 PMC1563146

[80] Johnson HW, Schell MJ. Neuronal IP3 3-kinase is an F-actin-bundling protein: role in dendritic targeting and regulation of spine morphology. *Mol Biol Cell* 2009;20:5166–80. 10.1091/mbc.e09-01-0083 19846664 PMC2793293

[81] Alfonso J, Fernández ME, Cooper B et al. The stress-regulated protein M6a is a key modulator for neurite outgrowth and filopodium/spine formation. *Proc Natl Acad Sci U S A* 2005;102:17196–201. 10.1073/pnas.0504262102 16286650 PMC1287971

[82] Ou M, Zhao M, Li C et al. Single-cell sequencing reveals the potential oncogenic expression atlas of human iPSC-derived cardiomyocytes. *Biol Open* 2021;10:bio053348. 10.1242/bio.053348 33589441 PMC7903994

[83] Miki K, Deguchi K, Nakanishi-Koakutsu M et al. ERRγ enhances cardiac maturation with T-tubule formation in human iPSC-derived cardiomyocytes. *Nat Commun* 2021;12:3596. 10.1038/s41467-021-23816-3 34155205 PMC8217550

[84] Long Q, Yang K, Yang Q. Regulation of mitochondrial ATP synthase in cardiac pathophysiology. *Am J Cardiovasc Dis* 2015;5:19–32. 26064790 PMC4447074

[85] Lee AS, Kranzusch PJ, Doudna JA et al. eIF3d is an mRNA cap-binding protein that is required for specialized translation initiation. *Nature* 2016;536:96–9. 10.1038/nature18954 27462815 PMC5003174

[86] Verma SK, Deshmukh V, Thatcher K et al. RBFOX2 is required for establishing RNA regulatory networks essential for heart development. *Nucleic Acids Res* 2022;50:2270–86. 10.1093/nar/gkac055 35137168 PMC8881802

[87] Gallanti A, Prelle A, Moggio M et al. Desmin and vimentin as markers of regeneration in muscle diseases. *Acta Neuropathol* 1992;85:88–92. 10.1007/BF00304637 1285499

[88] Cao J, Routh AL, Kuyumcu-Martinez MN. Nanopore sequencing reveals full-length tropomyosin 1 isoforms and their regulation by RNA-binding proteins during rat heart development. *J Cell Mol Med* 2021;25:8352–62. 10.1111/jcmm.16795 34302435 PMC8419188

[89] Yoon J, Jordon J, van der Schaar M . GAIN: missing data imputation using generative adversarial nets. In: Dy J, Krause A (eds.), Proceedings of the 35th International Conference on Machine Learning, vol. 80. Stockholm, Sweden: PMLR, 2018, 5689–98.

[90] Lopez R, Regier J, Cole MB et al. Deep generative modeling for single-cell transcriptomics. *Nat Methods* 2018;15:1053–8. 10.1038/s41592-018-0229-2 30504886 PMC6289068

[91] Eraslan G, Simon L, Mircea M et al. Single-cell RNA-seq denoising using a deep count autoencoder. *Nat Commun* 2019;10:390. 10.1038/s41467-018-07931-2 30674886 PMC6344535

[92] Li YI, Knowles DA, Humphrey J et al. Annotation-free quantification of RNA splicing using LeafCutter. *Nat Genet* 2018;50:151–8. 10.1038/s41588-017-0004-9 29229983 PMC5742080

[93] Rio DC . Reverse transcription–polymerase chain reaction. *Cold Spring Harb Protoc* 2014;2014:1207–16. 10.1101/pdb.prot080887 25368309

[94] Ho-Pun-Cheung A, Bascoul-Mollevi C, Assenat E et al. Reverse transcription-quantitative polymerase chain reaction: description of a RIN-based algorithm for accurate data normalization. *BMC Mol Biol* 2009;10:31. 10.1186/1471-2199-10-31 19368728 PMC2679744

[95] Bybee SM, Bracken-Grissom H, Haynes BD et al. Targeted amplicon sequencing (TAS): a scalable next-gen approach to multilocus, multitaxa phylogenetics. *Genome Biol Evol* 2011;3:1312–23. 10.1093/gbe/evr106 22002916 PMC3236605

[96] Sanger F, Nicklen S, Coulson AR. DNA sequencing with chain-terminating inhibitors. *Proc Natl Acad Sci U S A* 1977;74:5463–7. 10.1073/pnas.74.12.5463 271968 PMC431765

