# Supplementary material for: scASprofiler: profiling single-cell RNA splicing with a deep convolutional generative network
Source: Brief Bioinform. 2026 Sep 11;27(5):bbag497. doi: 10.1093/bib/bbag497 (PMC13563674; doi:10.1093/bib/bbag497)
Supplement: Supplementary file 1 [file supplementary_file_bbag497_corrected.docx]

**Fig S1 Computational scalability of scASprofiler across datasets.** Runtime and peak memory usage across datasets with different cell numbers. Left: runtime in minutes. Right: peak CPU RSS and GPU allocated memory in GB.

**Supplementary Table S1. Runtime, memory usage and convergence behavior of scASprofiler.**

| **Dataset** | **Cells** | **Junctions** | **Early-stopping epoch** | **Best val. MSE** | **Runtime (sec)** | **Runtime (min)** | **CPU RSS peak (GB)** | **CPU USS peak (GB)** | **GPU alloc. peak (GB)** |
| --- | --- | --- | --- | --- | --- | --- | --- | --- | --- |
| PRJEB15062 | 139 | 11,685 | 52 | 0.0063 | 232.29 | 3.87 | 1.98 | 1.98 | 7.03 |
| GSE85908 | 203 | 16,870 | 41 | 0.0196 | 321.57 | 5.36 | 2.42 | 2.41 | 9.94 |
| GSE75748 | 659 | 2,814 | 19 | 0.0172 | 161.93 | 2.70 | 1.50 | 1.49 | 2.93 |
| mouse_brain | 4,865 | 27,379 | 13 | 0.0038 | 2,636.63 | 43.94 | 13.31 | 13.32 | 16.04 |
| GSE130731_10x | 31,880 | 37,226 | 42 | 0.0168 | 8,056.20 | 134.27 | 111.05 | 111.77 | 54.19 |

Note: Runtime and memory usage were profiled on a workstation equipped with an NVIDIA A800 GPU. CPU RSS, CPU USS, and GPU allocated memory indicate peak values during model execution.

**Fig S2 t-SNE comparison of SCASL and scASprofiler under increasing dropout.** Columns show dropout levels (10%–70%); top row: SCASL; bottom row: scASprofiler. Each dot represents a cell colored by the ground-truth label. (**a)** PRJEB15062: iPSC and Endoderm. (**b)** GSE85908: iPSC, NPC, and MN. (**c)** GSE75748: differentiation time course (0h–96h).

**Fig S3 Imputation performance of scASprofiler across datasets and dropout frequencies. (a)** Radar plots comparing scASprofiler and SCASL on three datasets (GSE75748, GSE85908, PRJEB15062) across increasing dropout frequencies using clustering metrics (ARI, AMI, NMI, FMI, Homo, V-M, NE). (**b)** Density curves of measured versus imputed values at artificially masked positions across dropout frequencies. (**c)** Jensen–Shannon (JS) divergence between imputed and raw distributions at artificially masked positions across dropout frequencies.

**Fig S4 t-SNE visualization of label-control analyses.** Columns show different label settings: label-conditioned scASprofiler, no-label, shuffled-label, and splicing-label. Each dot represents a cell colored by the ground-truth label. (**a)** PRJEB15062: iPSC and Endoderm. (**b)** GSE85908: iPSC, MN, and NPC. (**c)** GSE75748: differentiation time course (0h–96h).

**Fig S5 Label-control analysis of clustering performance.** The heatmap shows clustering metrics under label-conditioned scASprofiler, no-label, shuffled-label, and splicing-label settings across PRJEB15062, GSE85908, and GSE75748. Metrics include ARI, AMI, NMI, FMI, V-measure, Homogeneity, and Normalized Entropy.

**Fig S6 t-SNE visualization under random AS-site ordering.** Columns show five independent random feature-order seeds before image reshaping. Each dot represents a cell colored by the ground-truth label. (**a)** PRJEB15062: iPSC and Endoderm. (**b)** GSE85908: iPSC, MN, and NPC. (**c)** GSE75748: differentiation time course (0h–96h).

**Fig S7 Random AS-site ordering stability of scASprofiler.** Clustering metrics under five independent random AS-site orderings in PRJEB15062, GSE85908, and GSE75748. Colored points denote individual random-order runs, and black points with error bars indicate the mean and variation across runs. Metrics include ARI, NMI, AMI, FMI, V-measure, Homogeneity, and Normalized Entropy. Lower values are better for Normalized Entropy, whereas higher values are better for the other metrics. The results show that scASprofiler is robust to AS-site reordering.

**Fig S8 Ablation and *k*-sensitivity analyses of scASprofiler.** (**a)** ARI degradation of ablation variants relative to the full model. (**b)** Metric-level contribution to performance degradation. **(c)** Metric profiles across ablation variants. **(d)** Sensitivity of ARI to different *k* values.

**Fig S9 t-SNE comparison across single-cell splicing methods.** t-SNE embeddings were generated from method-specific splicing representations in **(a)** PRJEB15062, **(b)** GSE85908, and **(c)** GSE75748. Cells are colored by annotated cell type or differentiation stage. SCSES results were not available for GSE85908. scASprofiler showed clearer separation of annotated cell populations or developmental stages across datasets.

**Fig S10 Clustering performance across methods and datasets.** Heatmaps summarize clustering metrics for scASprofiler and comparison methods in **(a)** PRJEB15062, **(b)** GSE85908, and **(c)** GSE75748. Metrics include ARI, AMI, NMI, FMI, V-measure, Homogeneity, and Normalized Entropy. Higher values indicate better performance for all metrics except Normalized Entropy, for which lower values are better. scASprofiler showed the most favorable overall performance across datasets.

**Fig S11 Comparison of scASprofiler with single-cell imputation methods.** t-SNE visualizations and clustering metrics comparing scASprofiler with SSNMDI and scRNMF across PRJEB15062 (**a, b**), GSE85908 (**c, d**), and mouse brain (**e, f**). In the t-SNE panels, each dot represents a cell colored by the ground-truth label.

**Fig S12 Sashimi plots (a)** Sashimi plot of a complex alternative splicing event in *BCLAF1*. (**b)** Sashimi plot of a complex alternative splicing event in *DHX9*. (**c)** Sashimi plot of a complex alternative splicing event in *DPPA4*.

**Fig S13 Examples where AS signals distinguish cell types, whereas gene-level expression shows weaker separation. (a)** Violin plots of AS probabilities for two DPPA4 splice junctions (DPPA4_1322 and DPPA4_1323) in iPSC versus endoderm. (**b)** Comparison of mean AS probabilities for representative marker junctions from the raw data and the mean expression of their corresponding genes (heatmap/color scale). (**c-d)** Single-cell gene-expression heatmaps for the imputed-derived gene set and the raw-derived gene set (cells ordered by cell type), showing weaker cell-type separation than AS signals.

**Fig S14 Marker AS events separate cell types better than gene expression. (a)** Heatmap of mean AS probabilities for representative marker AS events across cell types. (**b)** Heatmap of mean expression for the corresponding genes across cell types.

**Fig S15 Raw data derived marker AS events show weaker cell-type separation. (a)** Heatmap of marker AS events identified from raw data without imputation. **(b-c)** AS probability visualizations across cell types for the same AS events (raw data and imputed data). (**d)** Mean gene expression heatmap (raw data) for the corresponding genes. (**e)** Gene expression heatmap (raw data) for the corresponding genes.

**Fig S16 GO biological process (GOBP) enrichment of differentially spliced genes across** **various cell types.** GOBP terms are shown for differentially spliced genes across various cell types.

**Fig S17 Examples of relationships between gene expression and splice junction usage.** t-SNE feature overlays for TPM1 and VIM showing gene expression (top) and corresponding splice junction usage in imputed and raw data.

**Fig S18 DAS concordance under label-control settings.** Comparison of DAS events between label-conditioned scASprofiler and no-label (**a**), shuffled-label (**b**), or splicing-label (**c**) settings. Columns show DAS event overlap, effect-size consistency, significance consistency, and top-ranked DAS event overlap.

**Fig S19 Pseudo-bulk LeafCutter support for imputed DAS events.** (**a)** Support ratios of imputed DAS events at intron and DAS-group levels. (**b)** Counts of imputed DAS introns/groups and LeafCutter-supported matched introns/groups. (**c)** Distribution of exact-matched intron fractions across imputed DAS groups.

**Fig S20 Permutation-based assessment of imputed DAS events.** (**a)** Null distribution of DAS event counts under permuted labels compared with the true-label result. (**b)** p-value calibration plot for permuted-label and true-label DAS analyses.

**Fig S21 Random-seed robustness of imputed DAS detection.** (**a)** DAS event counts across five random seeds. (**b)** Recurrence of DAS events across seeds. (**c)** Pairwise Jaccard similarity between seeds. (**d)** Effect-size variability across seeds. (**e)** Top-N DAS event overlap across seeds. (**f)** Direction stability of recurrent DAS events.

**Fig S22 DAS concordance under random AS-site ordering.** (**a-e)** Comparison of DAS events between the default feature order and five random feature-order seeds. Rows indicate random-order seeds 1–5; columns show DAS event overlap, effect-size consistency, significance consistency, and top-ranked DAS event overlap.

**Fig S23 k-sensitivity analysis of clustering performance.** Columns show clustering metrics across different k values: ARI, AMI, NMI, FMI, V-measure, Homogeneity, and Normalized Entropy. Rows show **PRJEB15062**, **GSE75748**, and **GSE85908**. The dashed line indicates the default setting (k = 10).
